# Iron homeostasis and post-hemorrhagic hydrocephalus: a review

**DOI:** 10.3389/fneur.2023.1287559

**Published:** 2024-01-12

**Authors:** Shelei Pan, Andrew T. Hale, Mackenzie E. Lemieux, Dhvanii K. Raval, Thomas P. Garton, Brooke Sadler, Kelly B. Mahaney, Jennifer M. Strahle

**Affiliations:** ^1^Department of Neurosurgery, Washington University School of Medicine, Washington University in St. Louis, St. Louis, MO, United States; ^2^Department of Neurosurgery, University of Alabama at Birmingham School of Medicine, University of Alabama at Birmingham, Birmingham, AL, United States; ^3^Department of Neurology, Johns Hopkins University School of Medicine, Johns Hopkins University, Baltimore, MD, United States; ^4^Department of Pediatrics, Washington University School of Medicine, Washington University in St. Louis, St. Louis, MO, United States; ^5^Department of Hematology and Oncology, Washington University School of Medicine, Washington University in St. Louis, St. Louis, MO, United States; ^6^Department of Neurosurgery, Stanford University School of Medicine, Stanford University, Palo Alto, CA, United States; ^7^Department of Orthopedic Surgery, Washington University School of Medicine, Washington University in St. Louis, St. Louis, MO, United States

**Keywords:** germinal matrix hemorrhage, intraventricular hemorrhage, posthemorrhagic hydrocephalus, iron overload, choroid plexus, ependyma, ferroportin, iron transporters

## Abstract

Iron physiology is regulated by a complex interplay of extracellular transport systems, coordinated transcriptional responses, and iron efflux mechanisms. Dysregulation of iron metabolism can result in defects in myelination, neurotransmitter synthesis, and neuronal maturation. In neonates, germinal matrix-intraventricular hemorrhage (GMH-IVH) causes iron overload as a result of blood breakdown in the ventricles and brain parenchyma which can lead to post-hemorrhagic hydrocephalus (PHH). However, the precise mechanisms by which GMH-IVH results in PHH remain elusive. Understanding the molecular determinants of iron homeostasis in the developing brain may lead to improved therapies. This manuscript reviews the various roles iron has in brain development, characterizes our understanding of iron transport in the developing brain, and describes potential mechanisms by which iron overload may cause PHH and brain injury. We also review novel preclinical treatments for IVH that specifically target iron. Understanding iron handling within the brain and central nervous system may provide a basis for preventative, targeted treatments for iron-mediated pathogenesis of GMH-IVH and PHH.

## Introduction

Iron homeostasis is critical to a variety of neurodevelopmental processes. Iron deficiency has been linked to impaired myelination (1, 2), altered monoamine neurotransmitter synthesis (2), and reduced hippocampal neuronal metabolism (3) in neonatal rats. Conversely, brain iron overload can also be deleterious (4–10). Therefore, understanding the homeostatic mechanisms that maintain the delicate brain iron balance is important to better understand how we can preserve the neuronal developmental environment after peri- and neonatal iron-related pathology in the central nervous system.

In preterm infants, brain iron homeostasis can be dramatically disrupted by germinal matrix hemorrhage-intraventricular hemorrhage (GMH-IVH) when bleeding from the immature, fragile vascular network of the germinal matrix releases red blood cells (RBCs) which subsequently lyse and release the blood breakdown products iron, hemoglobin, and bilirubin into the germinal matrix (Grade I), ventricles of the brain (Grades II, III), and brain parenchyma (Grade IV). Prognosis and mortality after GMH-IVH are related to the extent of hemorrhage, with higher grades of GMH-IVH associated with the worst neurodevelopmental outcomes (11, 12). In addition, 30% of infants with high grade (Grades III and IV) GMH-IVH develop post-hemorrhagic hydrocephalus (PHH) (13), a progressive dilation of the cerebral ventricles that results in secondary brain injury and for which definitive surgical management is difficult in preterm neonates (14).

While the mechanisms of PHH and other neurological sequelae after GMH-IVH are not clear, the cytotoxic effects of free iron for inducing DNA damage and disrupting oxidative processes are well documented (15), and clinical studies have shown a higher proportion of infants with PHH to have CSF free iron after GMH-IVH compared to control subjects (16). Unbound iron can participate in the Fenton reaction, in which Fe^2+^ can be oxidized by hydrogen peroxide to form hydroxyl free radicals and Fe^3+^ (17, 18). These free radicals can subsequently oxidize numerous cellular targets, causing significant damage and cell death. However, how free iron is directly linked to the pathogenesis of PHH and other devastating neurological sequelae after high grade (Grades III-IV) IVH has not been fully elucidated.

Complicating efforts to understand and target iron overload-mediated brain injury following GMH-IVH, iron processing in the developing brain is not as well-understood as it is in the adult brain. Until recently, the primary cellular brain iron transporters reported in the neonatal brain were divalent metal transporter 1 (DMT1), transferrin receptor (TFR), and ferritin, iron transporters that are also involved in iron handling in other epithelial tissues like the intestine. Recent clinical studies have underscored the role of extracellular iron pathway proteins such as transferrin, ceruloplasmin, haptoglobin, and hemopexin that may have a role in CSF iron clearance after IVH (19, 20). Nevertheless, the number of cellular iron transporters characterized in the neonatal brain is small by comparison to the number of iron handling proteins described in adult brain and neonatal peripheral tissues. This qualitative review summarizes our current understanding of iron transport and homeostasis in the brain, as well as the developmental time course of iron pathway protein expression. We also review the role these proteins may have in mediating iron and blood breakdown product clearance after neonatal GMH-IVH.

## Brain iron transport and homeostasis

Iron exists in several different stable states within the human body. The availability of iron in different oxidative states makes it a prime player in intracellular metabolic processes essential to life. In the plasma, circulating iron is primarily bound to the iron binding protein transferrin in the form of Fe^3+^ (ferric) iron (21). Low (<1 μM) concentrations of non-transferrin bound iron (NTBI) can also be present in the plasma in either Fe^2+^ (ferrous) or ferric states bound to small organic molecules like citrate (22, 23). Within hemoglobin or myoglobin molecules, iron is much less accessible. In these heme-bound states, ferrous iron is contained within the center of protoporphyrin IX scaffolds (24). Once inside a cell, ferrous iron represents the labile and active pool of iron that is readily used in biological processes, whereas ferric iron is usually stored complexed to the iron-storage protein ferritin (25). Mitochondrial ferritin (FtMt) is one of three ferritins that are encoded separately by the human body (the other two are the cytosolic L and H subunits) and is primarily found in the mitochondria of metabolically active organs like the brain and testis (26, 27). Mitochondria require iron to support the biogenesis of iron–sulfur clusters and heme synthesis (28–30), however close regulation of mitochondrial iron levels is needed to protect the mitochondria from iron-mediated oxidative damage. FtMt is believed to play a role in maintaining this mitochondrial iron homeostasis (31). In addition to ferritin, cellular iron can also be stored in complex with hemosiderin, but the iron in hemosiderin is not as readily available for use. Excess hemosiderin deposits form after hemorrhage and are thought to result from hemoglobin phagocytosis and subsequent heme breakdown into iron and biliverdin (12).

It is important to note that the majority of our understanding of brain iron metabolism is derived from experiments conducted in adult animals. As many of the following cellular iron transport proteins and mechanisms remain largely under characterized in the neonatal brain, and the developmental timelines of the expression of major iron handling proteins in the brain are still not known, it will be crucial to verify these models of brain iron transport and homeostasis in fetal and neonatal animals to advance our understanding of iron-related pathology in the neonatal time period.

### Systemic iron absorption and metabolism

The total amount of iron in the body is primarily determined by dietary intake and uptake from the gut (32, 33). Enterocytes, epithelial cells that line the lumen of the intestines, mediate dietary iron absorption in the duodenum and proximal jejunum of the small intestine (34). Non-heme dietary iron exists predominantly in its ferric form and must be reduced to its ferrous form by the ferrireductase duodenal cytochrome B on the apical brush border of enterocytes before it can be absorbed (35). Other ferrireductases may also play a role in converting ferric to ferrous iron (36). Ferrous iron enters the enterocyte via DMT1 expressed on the apical membrane of enterocytes and leaves the enterocyte via ferroportin 1 (FPN1) expressed on the basolateral surface (37, 38). Exported iron is oxidized to its ferric form via a ferroxidase and complexes with a protein (ie. transferrin) or iron-binding small molecule (ie. citrate) to enter the plasma (35, 39).

When the body’s iron stores are sufficient, the liver peptide hormone hepcidin can inhibit iron export from the enterocyte by binding to and causing FPN1 internalization (25). This prevents additional iron entry into the plasma and drives intracellular storage of iron as ferritin (25). Ferritin within the enterocyte that is not used is excreted when enterocytes are sloughed off the intestinal mucosa at the end of their approximately 3-day lifespan (25).

### Brain iron import across the blood brain barrier

Under physiological conditions, iron is transported into the brain from the circulation through a series of highly regulated and coordinated steps. Early models of iron transport into the brain proposed that iron-bound transferrin can bind to luminal TfR on microvascular endothelial cells of the capillaries and choroid plexus (ChP) that comprise the blood brain barrier (BBB) before being endocytosed and released into the brain extracellular space (i.e., transcytosis) (40–43). In more recent models of receptor mediated endocytosis brought on in part by the identification of DMT1 on brain capillary endothelial cells (BCECs) that form the BBB, endothelial cell iron release is thought to be more nuanced, with endocytosed transferrin dissociating into ferric iron and apotransferrin in the acidified endosome (44). Ferric iron is reduced to ferrous (Fe^2+^) iron, which is then transported into the cytoplasm of the endothelial cell via DMT1, where it can either be stored intracellularly with ferritin or exported into the interstitial fluid via FPN1 when ferritin is saturated. The ferroxidase ceruloplasmin oxidizes Fe^2+^ back to Fe^3+^ (45), which recombines with apotransferrin to re-form transferrin in the interstitium where it can be taken up by glia and neurons. Transferrin-independent mechanisms of iron import into the brain may also exist, including but not limited to putative NTBI import via ferritin receptors (46, 47), however these mechanisms are not well-characterized.

#### Neuronal and glial iron uptake and homeostasis

Iron is present in neurons, astrocytes, microglia, and oligodendrocytes where it plays essential roles in cell respiration, neurotransmitter synthesis, myelination, DNA synthesis, and other cellular processes (1, 48–56). Studies in adult mice and rats have revealed several mechanisms by which these cells may take up iron from the interstitial fluid. Adult neurons, which express Tfr, can obtain iron from transferrin (57). *In vitro* studies have shown neurons also express ferrous iron transporters zinc regulated transporter and iron regulated transporter like protein 8 (Zip8) and Dmt1 at the cell surface to mediate NTBI uptake (57). Similarly, oligodendrocytes in the adult mouse brain express Tfr, and *in vitro* express the ferritin receptor T-cell immunoglobulin mucin domain 2 (Tim-2) to facilitate NTBI iron uptake via ferritin (48, 49). *In vitro*, microglia and astrocytes express Tfr and Dmt1 at the cell surface to facilitate iron uptake via transferrin and NTBI, respectively (58–60). There is comparatively less evidence for Tfr and Dmt1 expression on glia *in vivo* (61). Fpn1 has been detected in neurons and glia, but its expression varies by age and region (62–66).

### Role of hepcidin in brain iron homeostasis

In non-inflammatory conditions, hepcidin reduces brain iron load by inducing endothelial cell Fpn1 internalization and degradation when interstitial Fe^2+^ levels rise (67–70). Hepcidin upregulation in inflammation can cause deleterious effects due to its role in inducing Fpn1 internalization in neurons and glia, which in turn increases intracellular iron levels (71). Therefore, regulation of hepcidin is an important and potentially targetable axis of iron homeostasis. It is known that systemic hepcidin produced from the liver can cross the BBB to enter the brain (72), and that increases in brain and systemic hepcidin after hemorrhagic or ischemic parenchymal brain injury lead to increased iron in the brain (72, 73). However, studies in adult rodents have shown that brain iron metabolism is not drastically altered in mouse models in which liver hepcidin production is knocked out (72), suggesting there are additional brain-specific hepcidin regulation pathways.

*In vivo* studies in adult rats and mice and *in vitro* studies in rat and human cells have shown that the expression of hepcidin in the brain is controlled through several mechanisms including the interleukin-6/janus kinase 2/signal transducer and activator of transcription 3 (IL-6/JAK2/STAT3), bone morphogenic protein/s-mothers against decapentaplegic (BMP/SMAD), and CCAT enhancer binding (C/EBP) homologous protein (CHOP) pathways (70, 74–80). As a potential mechanism for the neurotoxic effects of hepcidin overexpression in inflammation, it is known that lipopolysaccharide (LPS) released during inflammation stimulates Toll-like receptor 4 (TLR4), a signaling pathway which (1) has previously been shown to underlie ChP-CSF interface inflammation in both post-infectious hydrocephalus and PHH (81), and (2) induces the production and release of critical cytokines like interleukin-6 (IL-6) (82, 83). IL-6 can upregulate hepcidin expression in the brain via the JAK2/STAT3 pathway (76, 77). The BMP/SMAD pathway has similar effects that are specific to hepcidin upregulation in microglia (76), and the CHOP pathway has been shown to play a role in pathology after subarachnoid hemorrhage (SAH) in adult rodents via its effect of upregulating hepcidin expression in neurons (84), thereby preventing iron export out of neurons, increasing neuronal iron content, and inducing apoptosis. Acute increases in brain iron load can also induce hepcidin upregulation (70).

### Post-transcriptional coordination of brain iron homeostasis

In addition to the bidirectional effects of hepcidin regulation on iron load in the brain, the iron-responsive element (IRE) signaling pathway and cytoplasmic iron regulatory proteins (IRP-1 and IRP-2) play a major role in maintaining brain iron homeostasis at the post-transcriptional level. IRP-1 and IRP-2 are iron and mRNA-binding proteins which can bind to IREs, relatively short and conserved hairpin-loops in the 3′ or 5′ untranslated region (UTR) of IRP target mRNA molecules (85–89). IRP-1 has additional functionality as a cytoplasmic aconitase in iron-rich conditions. Depending on where in the target mRNA the IRE is localized (90, 91), IRP binding can (1) block ribosome binding, translation, and synthesis of key iron pathway proteins including ferritin and Fpn1 (86, 92–97), or (2) stabilize the mRNA to increase the synthesis of iron pathway proteins like Tfr and Dmt1 (87, 98). Iron binding to IRPs decreases their affinity for IREs and induces their dissociation from mRNA molecules (87, 88, 99), offering a mechanism to control iron pathway protein synthesis that is responsive to iron levels in the brain.

## Disruptions in the homeostatic balance of brain iron after GMH-IVH

Hemorrhage in neonatal IVH most commonly originates from the immature blood vessels of the germinal matrix and is associated with significant morbidity and mortality in preterm infants (100). RBC lysis in the CSF after IVH releases blood breakdown products including bilirubin, hemoglobin, and unbound ferrous iron, the latter of which can be oxidized in the Fenton reaction to form cytotoxic hydroxyl free radicals and ferric iron (12). While iron homeostasis mechanisms keep free iron levels under control in physiologic conditions, IVH may release amounts of hemoglobin and free iron that overwhelm iron handling and clearance systems in the brain.

**Figure 1:**
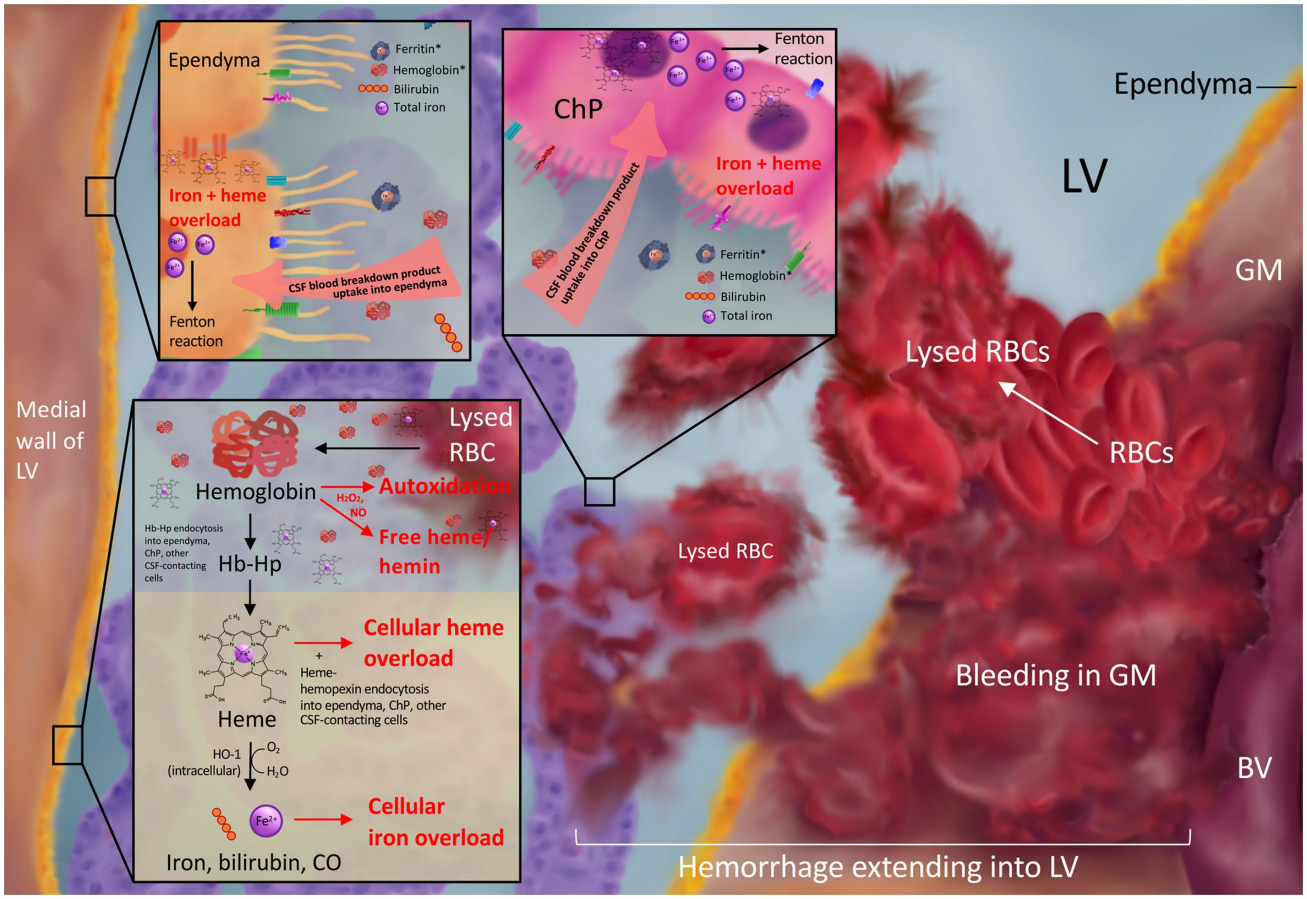
Blood breakdown product release into the CSF after intraventricular extension of germinal matrix hemorrhage. Bleeding from the ruptured immature blood vessels (BV) of the germinal matrix (GM) results in red blood cell (RBC) release into the GM and lateral ventricle (LV) and subsequent lysis to release blood breakdown products including hemoglobin, ferritin, bilirubin, and iron into the CSF. CSF hemoglobin and ferritin (asterisks in top panels) are elevated in the setting of post-hemorrhagic hydrocephalus. Subsequent ependymal and choroid plexus uptake of blood breakdown products from the CSF may lead to toxic iron and heme overload (top panel). RBC lysis in the LVs can release free hemoglobin into the CSF, which may undergo cytotoxic autoxidation in the presence of H2O2 and/or NO and release free heme/hemin into the CSF (bottom panel). Hemoglobin may also be bound and stabilized by the scavenger haptoglobin for cellular uptake and subsequent degradation into heme and Fe2+ ions leading to overload in the setting of GMH-IVH (bottom panel).

Free iron in the CSF may be particularly deleterious due to its free access to the ependymal cells that line the surfaces of the ventricles and the CSF-producing cells of the ChP (81), in addition to its proximity to the cells that make up neurodevelopmentally-important periventricular structures including the subventricular zone, white matter, and hippocampus (Figure 1). This may make these cells particularly susceptible to iron uptake and overload. In fact, previous research from Strahle et al. reported subventricular zone iron overload after IVH (101), and Garton et al. demonstrated iron-mediated cell death in hippocampal neurons in a rodent IVH model (102). This was consistent with studies conducted in humans showing perinatal brain injury is associated with smaller hippocampal size in preterm infants (103).

Other studies have shown intracellular iron accumulation within perihematomal neurons and glia after GMH in rodents (104); ependymal and subependymal hemosiderin deposition, ferritin expression, and iron accumulation after IVH (105, 106); as well as hemosiderin deposition, ependymal cell death, and subependymal damage in human neonates with IVH-PHH (107). It is also possible that free iron and hemoglobin released into the ventricles after IVH may be transported to distant intraparenchymal regions via the CSF (108), as intraventricular radioactive iron has been shown to distribute to distant anatomic areas of the brain parenchyma in neonatal rats (109).

## Iron transporters, scavengers, and related proteins in the neonatal brain

Recent studies have highlighted the role that the iron handling proteins/scavengers haptoglobin, hemopexin, and ceruloplasmin play in blood and blood breakdown product clearance in the neonatal brain after IVH (19, 20). In this section, we review the developmental time course of iron handling protein expression in the fetal and neonatal brain parenchyma and CSF (Supplementary Table 1). We start with extracellular iron transporters and scavengers in the serum including transferrin, haptoglobin, hemopexin, and ceruloplasmin. We then discuss the expression and localization of the cellular iron transporters ferritin, TFR, DMT1, FPN1, low-density lipoprotein receptor-related protein 1 (LRP1), CD163, and the heme oxygenases. We also discuss proteins that play other key roles in neonatal brain homeostasis, including hepcidin, the IRPs, and amyloid precursor protein (APP). Understanding the molecular mechanisms that mediate iron transport and metabolism in the neonatal brain is necessary to advance our understanding of pathologic iron overload after IVH that leads to inflammation, PHH, and other forms of immediate and delayed injury to the brain.

### Extracellular iron transporters and scavengers that have been identified in the neonatal brain

#### Transferrin

Transferrin is a glycoprotein that binds to and transports ferric iron through the circulation prior to intracellular delivery. Transferrin is primarily produced by liver hepatocytes, and transferrin-mediated delivery of iron accounts for the majority of iron transport into the brain from the circulation (110, 111). However, the observed rate of iron import into the brain is significantly higher than the rate of transferrin import across the developing BBB (112–114), suggesting there are additional brain endogenous transferrin production mechanisms.

Specifically, transferrin found in the interstitial fluid is produced by oligodendrocytes and the ChP of the lateral and third ventricles (115–118). Endogenous transferrin functions to rapidly bind imported ferric iron ions to mediate delivery to neurons and other cell types. Transferrin has also been shown to play role in oligodendrocyte maturation and enhancing myelinogenesis (119, 120). β-2 transferrin is a desialylated isoform of transferrin synthesized in the brain that is found in the CSF and perilymph only (121).

##### Developmental time course of transferrin expression in the brain

Brain transferrin levels peak at birth before declining over the first 2–3 postnatal weeks, stabilizing at postnatal day 24 (122–129), and remaining constant throughout the rest of life (113). This decline in mouse and rat brain transferrin levels is region-specific, with a faster rate of decline in the cortex and hindbrain compared to the midbrain (113). In contrast to other cell types, BCEC transferrin expression remains high throughout development (127).

In addition to parenchymal brain transferrin, there is transferrin in fetal rat CSF (130), with a three-fold increase in CSF transferrin from 12 days gestation to 22 days (birth) followed by a significant decrease by postnatal day 10 (130). CSF transferrin has also been detected in human fetuses (131). Transferrin is transported via the CSF to periventricular structures including the medial habenular nucleus, mamillary bodies, interpeduncular nucleus, and brainstem after intraventricular injection into the lateral ventricles of neonatal postnatal day 7 rats (109). The exact role of CSF transferrin in the neonatal brain is not well understood, but it may have a role in transporting iron throughout the developing brain via the CSF.

##### Transferrin after neonatal GMH-IVH

Mahaney et al. previously demonstrated in humans that there are no significant differences in CSF transferrin levels after low- and high-grade IVH compared to neonates without IVH (19). However, when Strahle et al. followed CSF iron pathway protein levels over time in a separate study, there was an association between longitudinal increases in ventricular CSF transferrin levels after neonatal IVH-PHH and improved cognitive outcomes at 2 years of age (20). Because CSF transferrin is typically fully saturated with ferric iron in physiologic conditions, the blood breakdown products and iron released into the CSF after IVH may overwhelm the iron-binding capabilities of endogenous transferrin. This may lead to high levels of free iron within the ventricular system and subsequent damage to periventricular structures like the hippocampus and white matter (16, 102, 103). Increases in CSF transferrin may thus represent an adaptive physiological mechanism that protects against further injury, however this warrants further investigation.

#### Haptoglobin

The hemoglobin-binding scavenger haptoglobin plays an important role in mediating iron recycling and clearance, preventing oxidative damage by sequestering hemoglobin, and facilitating other anti-inflammatory activities in both physiologic and pathologic conditions. Haptoglobin is primarily produced by liver hepatocytes as an approximately 85 kDa multimeric protein with two H chains which can each bind an alpha beta dimer of free extracellular hemoglobin to prevent autoxidation and oxidative tissue damage (132). The haptoglobin-hemoglobin complex is irreversibly stable and is cleared by CD163 receptor-mediated endocytosis followed by intracellular degradation in a variety of cells including macrophages, monocytes, and microglia (133–139). Recent studies in rodents have reported CD163 is upregulated on hippocampal neurons after intracranial hemorrhage (ICH) (140, 141). CSF haptoglobin has also been previously characterized in human neonates (142).

##### Developmental time course of haptoglobin expression in the brain

Haptoglobin is present in low quantities in human serum at birth before increasing to adult levels over the first year of life (143, 144). Low levels of haptoglobin synthesis have been identified in the human brain at various stages of fetal development, with the highest haptoglobin expression identified in neurons at 6–8 weeks’ gestation (145). These levels decrease to variable levels of expression at 9–22 weeks’ gestation before rising again at 25–36 weeks’ gestation (145). Compared to neurons, there is less haptoglobin expression within endothelial cells, with variable levels of haptoglobin from 6 to 10 weeks’ gestation that decrease to no expression after 14 weeks’ gestation (145). No haptoglobin was identified in glia at any fetal timepoints evaluated in this study (145).

In a separate study of human fetal brains from 10 to 18 weeks’ gestation, haptoglobin mRNA was not present in brain tissue until 14–18 weeks’ gestation (146). Qualitatively, the authors reported the highest immunopositivity for haptoglobin mRNA at the last timepoint studied (18 weeks) (146). Haptoglobin mRNA levels at postnatal timepoints were lower than those observed during fetal development with regional differences across the basal ganglia, hypothalamus, and cortex (146).

##### Haptoglobin after neonatal GMH-IVH

The role of haptoglobin in the context of hemoglobin scavenging after neonatal IVH is still being elucidated. *In vivo* studies in rabbits have demonstrated that intraventricular injection of haptoglobin attenuates hemoglobin-induced inflammation, cytotoxicity, and structural damage (147). *In vitro* experiments incubating ChP cells from human neonates with IVH in haptoglobin recapitulate these results (147).

Mahaney et al. previously reported that there are no significant differences in CSF haptoglobin levels between human neonates with and without IVH (all grades) or PHH (19). In conjunction with a study showing haptoglobin expression in the cord blood of premature neonates in response to inflammation is associated with decreased risk of IVH and cerebral palsy (143), these results may suggest that haptoglobin-mediated hemoglobin scavenging mechanisms are exhausted after IVH and that upregulating brain haptoglobin may be a potential target to explore to prevent hemoglobin-mediated neurotoxicity (143). Alternatively, elevated haptoglobin may allow for increased haptoglobin-hemoglobin complex formation and subsequent internalization and degradation into heme and iron in CD163-expressing brain cells, leading to iron overload.

#### Hemopexin

Hemopexin is primarily produced by the liver and then released into the plasma. Analogous to haptoglobin binding of hemoglobin, hemopexin scavenges and binds to heme, a molecule composed of a ferrous iron ion coordinated to a porphyrin ring. Also known as Fe^2+^ protoporphyrin IX, heme is a precursor to hemoglobin and comprises the non-protein component of hemoglobin. Hemopexin-bound heme is an important co-factor involved in a variety of physiological processes in the brain including neuronal differentiation, growth, and survival (148–152).

Like most iron-containing compounds, heme can also have potentially deleterious effects on surrounding tissues when not bound to hemopexin or other hemoproteins. Free heme can be released from unbound, oxidized hemoglobin in times of haptoglobin saturation and subsequently participate in Fenton reactions. Hemopexin plays a role in blocking heme’s pro-oxidative activity and facilitating cellular heme uptake via CD91/LRP1 (153).

##### Developmental time course of hemopexin expression in the brain

There have been several reports of hemopexin synthesis in neurons and ependyma in the adult human and mouse brain (154–157), however the developmental time-course of hemopexin expression patterns in the parenchyma of the neonatal and postnatal brain is not well-defined. In the human fetal brain, hemopexin protein is expressed in neurons from 6 to 36 weeks’ gestation (145). Hemopexin mRNA has not been identified in the human fetal brain from 10 to 18 weeks’ gestation (146).

##### Hemopexin after neonatal GMH-IVH

Hemopexin has been identified in the neonatal and adult human CSF in physiologic and pathologic states including Alzheimer’s disease (158), diffuse large B cell lymphoma (159), degenerative disk disease (160), SAH, and IVH (19, 161). Mahaney et al. previously reported CSF hemopexin is not elevated after IVH-PHH in human neonates, and CSF hemopexin levels are positively correlated with ceruloplasmin and transferrin levels after IVH-PHH (19). In a separate study in human neonates, Strahle et al. also reported CSF hemopexin is the only iron scavenger that increased between temporary and permanent CSF diversion, and that ventricle size after IVH was inversely correlated with CSF hemopexin levels (20). In the context of previous studies showing the induction of hemopexin expression improves outcomes after ICH in mice (162), and elevated CSF hemopexin is predictive of poor neurological outcomes after SAH in adult humans (163), understanding the role of hemopexin in heme scavenging after IVH represents a pertinent potential therapeutic and diagnostic direction.

#### Ceruloplasmin

Ceruloplasmin is a ferroxidase that oxidizes ferrous iron ions to their ferric form, which is then bound by transferrin. Within the brain, ceruloplasmin is thought to play a critical role in facilitating both cellular iron export (via FPN1) and import to maintain iron homeostasis (164–170). Because ceruloplasmin produced in the liver cannot cross the BBB in significant quantities (171), cells in the adult and neonatal brain, ChP, and retina produce an alternatively spliced form of glycosylphosphatidylinositol (GPI)-anchored ceruloplasmin which comprises the majority of brain endogenous ceruloplasmin (172–174).

##### Developmental time course of ceruloplasmin expression in the brain

In 1988, Møllgård et al. reported ceruloplasmin mRNA expression in the human fetal brain from 14 to 36 weeks’ gestation (145). The protein was identified in neurons starting at 14–18 weeks’ gestation all the way through 36 weeks’ gestation (145). Weak ceruloplasmin expression was also reported in glia from 14 to 22 weeks’ gestation (145).

A more recent study of ceruloplasmin expression in the fetal mouse brain reported GPI-anchored ceruloplasmin protein appeared at embryonic day 12.5 (175), while diffusible ceruloplasmin (defined as ceruloplasmin secreted from the liver and/or released by the GPI anchor) was not detected until embryonic day 17.5 (175). GPI-anchored and diffusible ceruloplasmin levels increased from the time they were initially detected until postnatal day 1, before plateauing at postnatal day 7 and then subsequently decreasing (175). GPI-anchored ceruloplasmin expression on the surface of astrocytes has separately been reported in postnatal day 3 rats (174), however the timeline of postnatal cell-specific and regional expression patterns is not otherwise well-defined.

While low in concentration relative to serum levels, ceruloplasmin has also been reported in fetal and neonatal CSF in humans (6.57 ± 3.53 μg/mL in CSF vs. 20–130 μg/mL in serum) (19, 171, 176). No significant differences in CSF ceruloplasmin concentration over the course of gestation were identified, however sex differences in CSF ceruloplasmin were identified with male fetuses having higher CSF ceruloplasmin concentration than females (171).

##### Ceruloplasmin after neonatal GMH-IVH

Mahaney et al. previously reported no significant differences in CSF ceruloplasmin in humans after neonatal IVH-PHH when compared to control neonates (19), however there were correlations in CSF ceruloplasmin with CSF transferrin and hemopexin. Strahle et al. also reported no significant changes in CSF ceruloplasmin between temporary and permanent CSF diversion in human infants with PHH (20). The role of ceruloplasmin and other ferroxidases in brain iron metabolism throughout development merits further investigation.

### Membrane iron transporters and scavengers that have been identified in the neonatal brain

#### Transferrin receptor

Expressed on the apical cell surface, the dimeric transmembrane glycoprotein TFR mediates cellular uptake of ferric iron by binding to its ligand, transferrin, in a pH-dependent and reversible manner (177). The transferrin-TFR complex is internalized by receptor-mediated endocytosis, and subsequent acidification of the endosome causes transferrin to release its ferric iron for transport into the cytosol of the cell (61). Apotransferrin has a high affinity for TFR at low pHs, and they are together recycled back to the plasma membrane via exocytosis.

##### Developmental time course of transferrin receptor expression in the brain

To facilitate peripheral transferrin-mediated brain iron import, developing BCEC in mice express Tfr from the time they differentiate (178), with the peak in Tfr expression on rat BCECs occurring around postnatal week 2 between postnatal day 10 and postnatal day 21 (179). This coincides with the increase in iron import into the rat brain during the first two postnatal weeks in addition to rapid brain development and growth (180). Tfr expression in BCEC and neurons is also increased in iron deficient conditions in rats (41, 180).

The timeline of Tfr expression on other cells within the brain is distinct from that of BCEC Tfr because while variable low levels of Tfr expression in the neonatal rat ependyma, glia, and cerebral cortex and striatal neurons have been demonstrated as early as postnatal day 5 (181), and Tfr expression has been reported in the rat medial habenula at birth (179), robust and substantive Tfr expression is not seen in until at least postnatal day 15 in rats (181). The peak expression of neuronal Tfr does not occur until postnatal weeks 3–4 after reaching a plateau around postnatal day 21 (179). ChP epithelial cells and hippocampal neurons in rats display virtually no Tfr expression at postnatal days 5 and 10, however become strongly positive by postnatal day 15 (181).

The delayed peak in Tfr expression on rat neurons and glia until at least the third postnatal week has previously been hypothesized to be physiologically linked to the decrease in iron import into the brain from the circulation around the same time and the onset and increase of oligodendrocyte transferrin synthesis during postnatal days 10–25 (118, 179, 180). In conjunction with data showing that iron deficiency enhances brain Tfr expression during all ages (179), it is possible that Tfr expression serves as a compensatory mechanism for neurons and other brain cell types to maintain iron uptake necessary for development and function as overall brain iron levels decrease.

Tfr expression after neonatal GMH-IVH has not been extensively explored and merits further investigation.

#### Divalent metal transporter 1

DMT1 is a proton-coupled divalent metal ion symporter found in the plasma membrane and endosomes of various cell types in the body (61). In the brain, DMT1 is best known for its role in cellular iron uptake by transporting non-heme ferrous iron from acidified endosomes into the cytosol of neurons after receptor-mediated endocytosis of transferrin from the brain interstitium (182). While there have been several conflicting reports of DMT1 expression on non-neuronal cell types in the brain *in vivo* (37, 61, 181, 183, 184), DMT1 is now generally accepted to be expressed in low levels in BCEC endosomes to facilitate brain iron uptake across the BBB via receptor-mediated endocytosis of transferrin (182). Dmt1 has also been identified in developing rat ChP epithelial cells and glia including astrocytes and oligodendrocytes (181), however it is likely that there are other non-Dmt1-dependent mechanisms for iron uptake in these cells (61). Dmt1 has also been implicated as a mediator of ferroptosis after brain hemorrhage in rats (185).

First identified in mice in 1995 as the natural resistance-associated macrophage protein 2 (Nramp2) before its functional characterization as the proton-coupled metal-ion transporter divalent cation transporter-1 (Dct-1) in 1997 (37, 186), DMT1 was later understood to have 4 isoforms encoded by the Solute Carrier Family 11-member 2 (SLC11A2) gene that are now known to be differentially expressed across organs and within organelles. Two isoforms have alternative transcripts differing in the 3’ UTR, where one contains an iron response element (Type 1, +IRE) and the other (Type 2, -IRE) does not (187). The third (1A) and fourth (1B) isoforms differ in mRNA processing in the 5′ end, where the 1A transcript starts in Exon 1A, which is upstream of Exon 1B where the 1B isoform transcript starts, and skips over Exon 1B to be spliced directly to Exon 2 (188).

##### Developmental time course of divalent metal transporter 1 expression in the brain

*Dmt1* mRNA expression in the neonatal rat brain has been reported as early as postnatal day 3 (189), with the highest regional expression localized to the corpus callosum and on Purkinje cells of cerebellum (190). A separate study identified +IRE mRNA expression and low-level -IRE *Dmt1* mRNA expression in the cortex, hippocampus, striatum, and substantia nigra in postnatal week 1 rats (191), which increased in all regions by postnatal week 3 (191). +IRE *Dmt1* mRNA subsequently decreased from postnatal weeks 3–28 in the cortex, hippocampus, and striatum, but increased in the substantia nigra (191). Over the same developmental time period, -IRE *Dmt1* mRNA increased or stayed relatively constant in the rat hippocampus, striatum, and substantia nigra, but decreased in the cortex (191).

In a study evaluating cellular Dmt1 expression at postnatal days 5, 10, and 15, high levels of Dmt1 expression (isoform unspecified) were identified in blood vessels and ependymal cells at all three timepoints (181). Variable Dmt1 expression was identified in rat ChP epithelial cells from postnatal day 5 to 10 before turning into more robust expression by postnatal day 15 (181, 183). Dmt1 expression in glia followed a similar developmental time course (181, 183). Similarly, neuronal Dmt1 expression was variable at postnatal days 5 and 10 before increasing by postnatal day 15, with slight variations by brain region in the earlier timepoints (183).

Together, these studies indicate low-level Dmt1 expression in the neonatal rat brain that substantially increases over the first 2–3 weeks of rat postnatal development. This timeline mirrors developmental changes in brain iron and transferrin uptake from the circulation, which increase over the first 2–3 postnatal weeks (180), in addition to the peak in TFR expression on brain ECs between postnatal days 10–21 (179).

#### Ferroportin 1

Ferroportin 1, also known as solute carrier family 40 member 1 (SLC40A1), metal transporter protein 1 (MTP1), and iron-regulated transporter 1 (IREG1), is a transmembrane ferric iron transporter protein and the only known iron transporter responsible for cellular iron export (192). Initially characterized as an iron export protein on the basolateral surface of duodenal enterocytes (193), the basal surface of placental syncytiotrophoblasts (38), and the cytoplasmic compartment of reticuloendothelial cells (96), Fpn1 has more recently been implicated in transporting iron across the abluminal membrane of endothelial cells that comprise the BBB, depositing excess iron into the brain interstitial fluid when ferritin stores are replete (66).

##### Developmental time course of ferroportin 1 expression in the brain

Although the majority of studies of brain Fpn1 localization and function have been performed in adult rodents (63, 194), several studies have reported Fpn1 expression in various cell types across several regions of the developing brain. A 2001 paper by Burdo et al. mentions Fpn1 expression in the embryonic rat central nervous system (195), and a study by Yang et al. describes its expression in BCEC at postnatal day 0 with decreasing expression through postnatal week 8 (196). Fpn1 expression has also been identified in the soma, dendrites, and axons of cortex, striatum, hippocampus, midbrain, brainstem, and cerebellar neurons from postnatal day 7 to 70 (65), with the highest regional expression in the hippocampus (64). Fpn1 levels in the brain generally increase with age, with the lowest expression seen at postnatal week 1 followed by a progressive increase to postnatal week 9 with subsequent declines out to postnatal week 28 (64). Fpn1 has also been identified in postnatal oligodendrocytes and Schwann cells (65, 197).

##### Ferroportin 1 after neonatal GMH-IVH

FPN1 expression has not been studied in the context of neonatal IVH-PHH, however previous investigations have reported decreased Fpn1 expression in the cortex and hippocampus after SAH (198). Injection of hepcidin, a hormone that regulates Fpn1 expression by inducing Fpn1 internalization, further reduced Fpn1 levels after SAH leading to cytotoxic cellular iron overload (198). A separate study demonstrated Fpn1 upregulation in perihematomal brain tissue after intracerebral hemorrhage (199). Experimentally knocking out Fpn1 expression in the striatum with stereotaxic AAV-Cre injection in a Fpn-floxed mouse model significantly worsened iron-related pathology and neurologic outcomes after ICH induction (199). The role of FPN1 in attenuating cellular iron overload after neonatal IVH should explored as a potential therapeutic target.

#### Low-density lipoprotein receptor-related protein 1

LRP1, also known as CD91, is a transmembrane heme, hemopexin, and heme-hemopexin complex receptor protein (200). Beyond its roles in mediating heme clearance, LRP1 is also involved in tissue-specific functions including intracellular signaling (201), cell migration (202–204), lipid homeostasis (205–208), and protein scavenging (209). Of LRP1’s CNS-specific functions, two of the most widely studied are BBB regulation and amyloid-beta trafficking clearance (209–211).

##### Developmental time course of low-density lipoprotein receptor-related protein 1 expression in the brain

Lrp1 expression has previously been reported in mature neurons in the hippocampus, cortex, and cerebellum of the adult rat brain (212, 213), as well as radial glia in the embryonic mouse brain (214). More recently, Lrp1 expression in the CNS was reported to vary by age and cell type (215). In a 2016 study of Lrp1 expression in the embryonic day 13.5 to postnatal day 60 mouse brain, total Lrp1 was reported to peak during postnatal development with stable expression in radial glia, neuroblast, microglia, astrocytes, and neurons through development and adulthood (215). Specifically, Lrp1 was highly expressed in radial glia at embryonic days 13.5–18, astrocytes at postnatal day 5, microglia at embryonic day 13 through postnatal day 60, neuroblasts at embryonic days 13.5–18, and neurons at postnatal day 5-adulthood (215). The proportion of oligodendrocyte precursor cells expressing Lrp1 increases dramatically across embryonic and postnatal brain development, with approximately 69% of oligodendrocyte precursor cells expressing Lrp1 at embryonic day 15.5 and ubiquitous expression in adulthood (215).

##### Low-density lipoprotein receptor-related protein 1 after neonatal GMH-IVH

While LRP1 expression in the brain is not well-characterized in the context of IVH, Lrp1 upregulation has been reported after ICH (200). Lrp1 has previously been shown to clear heme-hemopexin complexes after ICH (216), and prophylactic intraventricular administration of recombinant human LRP1 protein 20 min before ICH induction in adult mice led to a reduction in hematoma volume, BBB permeability, and other brain injury (216). Lrp1 has also separately been shown to attenuate white matter injury after SAH in rats (217). These results suggest LRP1 has neuroprotective effects, potentially by preventing heme overload and heme-mediated cytotoxicity and deserves further investigation as a therapeutic target after neonatal IVH.

#### CD163

CD163 is a high-affinity scavenger receptor that mediates endocytosis and internalization of the haptoglobin-hemoglobin complex from extracellular spaces including the interstitial fluid (138). While CD163 expression was initially thought to be restricted to macrophages and monocytes, CD163 is expressed in neurons and upregulated after exposure to hemoglobin (102, 140, 218). Soluble CD163 (sCD163) sheds from CD163-positive cells to circulate in the serum and CSF. Subsequent studies have reported that sCD163 scavenges intrathecal hemoglobin-haptoglobin complexes after SAH in adult humans (219).

In addition to its role in scavenging hemoglobin by way of binding and taking up the hemoglobin-haptoglobin complex, CD163 is also involved in anti-inflammatory signaling in macrophages (220). CD163 expression can induce interleukin-10 (IL-10) secretion after binding haptoglobin-hemoglobin complexes containing specific haptoglobin genotypes, which in turn promotes heme oxygenase-1 (HMOX-1) synthesis (221). IL-10 has also been shown to upregulate CD163 expression on monocytes and macrophages (222, 223), while proinflammatory markers like interferon-ɣ are known to decrease expression (223).

##### CD163 after neonatal GMH-IVH

Whereas CD163 likely serves an anti-inflammatory role in macrophages, neuronal CD163 may play a deleterious role after ICH and IVH (140, 218, 224). Because hemoglobin is cytotoxic to neurons, which lack key iron sequestering machinery present in macrophages including widespread HMOX-1 induction in response to hemorrhage (106, 225), increased haptoglobin-hemoglobin complex uptake by way of CD163 upregulation post-hemorrhage may lead to iron overload and neuronal cell death.

Consistent with this hypothesis, rat hippocampal neurons expressing CD163 co-localize with phosphorylated-Jun N-terminal kinase (p-JNK) after neonatal IVH (102). As p-JNK is a kinase that plays a key role in the apoptosis cascade, this suggests a mechanism by which CD163 facilitates excessive hemoglobin influx into neurons after IVH to result in cellular ferrous iron overload, oxidative damage, and ultimately cell death (218).

##### Six-transmembrane epithelial antigen of the prostate proteins

The six-transmembrane epithelial antigen of the prostate (STEAP) family of proteins is comprised of metalloreductases, among which several are ferrireductases that reduce ferric iron to its ferrous state. Of the four identified members of the Steap family (Steap1, Steap2, Steap3, Steap4), Steap2 is arguably best characterized in the brain, where it has been reported in hippocampal neurons and the embryonic mouse ChP (57, 226). At postnatal day 7, Steap2 expression is present in the cerebellar Purkinje cells, and within the superior colliculus, olfactory bulb, and various other anatomic regions in the mouse brain (227). Steap3 was first identified in erythroid precursors where it localizes to endosomes to facilitate transferrin-bound iron uptake (228), however it has also been reported in the lumbar dorsal root ganglion (229, 230). Steap1 and 4 are expressed more ubiquitously, with additional Steap1 upregulation in the prostate and Steap4 enrichment in the bone marrow, placenta, fetal liver, and adipose tissue (226). Within cells, all four Steap proteins partially co-localize with transferrin and TFR, with Steap2 showing the highest degree of co-localization (226).

It is not clear what role the STEAP family of proteins may have in iron homeostasis after intracerebral or intraventricular hemorrhages. STEAP expression and localization in relation to brain iron levels and the specific role of STEAP proteins in the setting of brain iron overload has not yet been evaluated and merits further investigation.

##### T cell immunoglobulin and mucin domain containing proteins

The TIM family of proteins are receptors for H ferritin that mediate ferritin uptake into cells. Tim-2, the rodent ortholog of the human TIM-1 receptor, has been identified on oligodendrocytes as the primary mechanism of oligodendrocyte iron uptake by way of ferritin endocytosis (48, 49, 231, 232). In addition to oligodendrocytes, Tim-2 expression has been identified in neurons, astrocytes, and microvasculature in the mouse brain and is seen at postnatal day 7, 14 and 22 (233).

### Intracellular iron transporters and scavengers that have been identified in the neonatal brain

#### Ferritin

Ferritin is a major iron storage protein that can be found both intracellularly, where it functions as a ferroxidase to sequester ferrous iron, and extracellularly in the cerebrospinal fluid and serum. Once ferrous iron enters a cell, ferritin binds and uses its di-iron ferroxidase centers to convert it to the ferric form for storage in the ferritin mineral central cavity (234–240). Iron can be subsequently released from ferritin in response to metabolic demand (241).

Ferritin can hold up to 4,500 iron atoms. Early buffer extraction studies showed that about 1/3 of non-heme iron in the brain is stored as ferritin (242). Subsequent reports have estimated that this figure could be as high as 90% (242–244). Therefore, ferritin is a key player in maintaining iron homeostasis by regulating the labile iron pool within the brain.

##### Developmental time course of ferritin expression in the brain

A developmental study of iron handling proteins in rats showed ferritin levels in the brain peak on postnatal day 2 before decreasing over the course of the first 2 postnatal weeks (113). This downward trend is reversed by postnatal day 17 when brain ferritin levels begin to rise again before stabilizing at high levels similar to those seen at postnatal day 2 by postnatal week 11 (113). 200–500% increases in ferritin levels occur between postnatal day 17 and 2 years, with the most dramatic increases seen in the cortical, pontine, and cerebellar regions (113).

On a cellular level, while one study reported that ferritin mRNA is exclusively found in neurons in the postnatal rat brain, the ferritin protein is in multiple cell types throughout the postnatal brain with differential localization of two functionally distinct ferritin subunits (H and L) across cell types (245). H ferritin, which has redox potential, was identified in oligodendrocytes in postnatal day 21 rats while L ferritin was found in oligodendrocytes, microglia, and astrocytes (245). Neurons express both H and L ferritin (245).

Other studies in adult non-human primates have shown that neurons predominantly express H ferritin (246), and that astrocyte L ferritin expression is primarily localized to the corpus striatum (247). In human fetuses, ferritin has been reported in glia from 6 to 36 weeks’ gestation, with an increase at 19–22 weeks’ gestation (145). More recently, FtMt has been identified in humans as a ferritin that exists only in the mitochondria and whose transcription is not dependent on iron unlike cytosolic ferritins (248). In the context of neurodevelopment, studies in rats have characterized the majority of ferritin-containing cells at postnatal day 5 as microglia, while the majority at postnatal day 30 are oligodendrocytes (247).

##### Ferritin after neonatal GMH-IVH

In additional to intracellular ferritin within cells in the parenchyma, extracellular CSF ferritin is a valuable indirect measure of brain iron load and has previously been considered as a potential marker for pathology related to Alzheimer’s Disease (249–252), SAH (253), glioblastoma (254), meningitis (255, 256), amyotrophic lateral sclerosis (257), and other disease processes (258).

Specific to IVH-PHH, Strahle et al. recently reported in neonatal humans that longitudinal decreases in CSF ferritin levels between temporary and permanent CSF diversion after PHH are associated with improved scores on cognitive and motor aspects of the Bayley III examination at 2 years of age (20). Larger ventricle size at the time of permanent CSF diversion was also associated with higher levels of CSF ferritin (20). Mahaney et al. also previously showed that elevated CSF ferritin levels were associated with early and severe ventriculomegaly after IVH-PHH (259). These data, in addition to rodent studies showing increases in ferritin positive cells in the hippocampus and periventricular area after IVH, may represent a compensatory increase in ferritin that is reflective of ferritin’s function in preventing iron toxicity in times of overload (260). Specifically, ferritin levels may increase to prevent ferrous iron released into the ventricles after IVH from reacting with H_2_O_2_ and producing cytotoxic hydroxyl radicals in the Fenton reaction. Alternatively, elevated CSF ferritin may be secondary to inflammation.

#### Heme oxygenase-1 and 2

HMOX-1 and HMOX-2 are two of three isoforms of heme oxygenase, an antioxidant enzyme that catalyzes the rate-limiting step of heme degradation. In contrast to the constitutively expressed HMOX-2 that is found in high levels in the brain, HMOX-1 expression in the adult CNS is low at baseline and must be induced by its substrate, heme, and/or a variety of other oxidative stimuli that cause cellular stress (261, 262). Once induced, HMOX-1 degrades heme using NADPH, O_2_, and cytochrome p450 reductase in a series of three monooxygenation cycles to create the molecule biliverdin in addition to the byproducts carbon monoxide and ferrous iron. Biliverdin can subsequently be converted to bilirubin, a potent antioxidant with anti-inflammatory properties. Ferrous iron can be stored within ferritin, and carbon monoxide can participate in downstream cytoprotective signaling cascades. HMOX-2 degrades heme by the same mechanism.

##### Developmental time course of heme oxygenase expression in the brain

While robust Hmox-2 expression has been reported in neurons, glia, and blood vessels, Hmox-1 expression in adulthood is limited to hippocampal and olfactory neurons in relatively low levels (263). Using a transgenic *Hmox-*1-*luciferase* reporter mouse, *Hmox-1* transcription in postnatal day 1 cerebral cortex was shown to be higher than in adult mice (264). *Hmox-1* transcription decreases through postnatal days 2–14 before reaching levels similar to those seen in adult mice (264). Similarly, Hmox-1 mRNA and protein levels are highest at postnatal days 1 and 3 before steadily declining over the perinatal time period (264). While *Hmox-2* transcription was not been measured *in vivo*, Hmox-2 mRNA and protein levels in the developing mouse cortex remain relatively constant from embryonic day 14 to postnatal week 6 (264).

##### Heme oxygenase after neonatal GMH-IVH

As the HMOX-catalyzed heme degradation pathway is one of the only mechanisms for cytotoxic heme removal from cells, HMOX-1 and HMOX-2 likely play essential roles in mediating iron clearance in a variety of iron overload-related pathologies including ICH, IVH in adults after hemorrhagic stroke, and neonatal GMH-IVH. In the context of these diseases, there has been a focus on HMOX-1 as it can be induced by its substrate heme, which is released from hemorrhage (265). Specifically, Hmox-1 is highly expressed in vasculature, microglia, and macrophages adjacent to the area of hemorrhage following ICH induction via collagenase VII-S injection into the caudate putamen in adult mice (266). Iron accumulation in a rat model of adult IVH (identified as T2* MRI hyperintensities) has also been associated with Hmox-1 upregulation (267). Hmox-1 expression in periventricular areas and the hippocampus and cortex is also increased 24 and 72 h after neonatal GMH-IVH in mice (101). *In vitro* evidence in cells derived from humans, mice, and rats, and *in vivo* experiments in rodents show that HMOX-1 has regional neuroprotective effects when experimentally upregulated via pharmacologic induction in neurons (268–277), which have physiologically high and ubiquitous Hmox-2 expression but low Hmox-1 expression. Together with HMOX-1’s role in catalyzing heme degradation, these findings may suggest HMOX-1 has a specific function to attenuate heme-related damage after brain hemorrhage and protect against subsequent cellular heme/iron overload.

### Other proteins important in neonatal brain iron homeostasis

#### Hepcidin

Hepcidin is a peptide hormone that is primarily produced in the liver. Through binding to and inactivating (via inducing internalization) the cellular iron exporter Fpn1 (278), hepcidin regulates intracellular iron load. While hepcidin synthesis in the brain remains somewhat controversial, studies in adult animals have reported hepcidin protein and/or mRNA expression across the olfactory bulbs, cortex, ChP, corpus callosum, subventricular zone, and hippocampus of rat, mouse, and human brains (279–282).

The role of hepcidin in the neonatal brain is even less well characterized, where developmental changes in hepcidin expression are poorly understood beyond a general increase in hepcidin mRNA from postnatal week 1 to adulthood in the murine cortex, striatum, and hippocampus (281). Strahle et al. previously reported hepcidin in the CSF after neonatal IVH in humans (20), with no changes in CSF hepcidin levels between temporary and permanent CSF diversion for PHH.

#### Iron regulatory proteins 1 and 2

IRP-1 and IRP-2 carry out post-transcriptional regulation of cellular iron uptake, storage, and efflux by binding to IREs within the UTRs of mRNAs that code proteins which play crucial roles in brain iron homeostasis. The IREs within the mRNA of the iron exporter *FPN1* and the iron storage proteins ferritin H and ferritin L are located in the 5’-UTR, and IRP binding during iron-deficient conditions functions as a post-transcriptional control by inhibiting translation (38, 96, 193, 283–285). *TFR* and *DMT1* mRNA contain IREs within the 3’-UTR (37, 88, 98, 286–288), and IRP binding helps protect the mRNA against endonuclease degradation to promote translation of the proteins to facilitate cellular iron uptake (289, 290). In physiological iron replete conditions, IRPs dissociate from IREs (291, 292), allowing ferritin and Fpn1 translation and *TFR* and *DMT1* mRNA degradation.

##### Developmental time course of iron regulatory protein expression in the brain

Over the course of neurodevelopment, variable Irp-1 and Irp-2 expression has been identified in the neonatal rat brain across ChP epithelia, ependyma, blood vessels, glia, and neurons as early as postnatal day 5 (181), with robust expression of both proteins by postnatal day 15 (181). The percentage of cells with IRP expression varies by anatomic brain region across different ages (181). *IRP2* mRNA has been identified in human fetal brain tissue, where it is present in significantly greater quantities than *IRP1* mRNA (293).

##### Iron regulatory protein after neonatal GMH-IVH

In the context of neonatal GMH-IVH, Irp2 expression has been reported to decrease 1–5 days after neonatal GMH-IVH induced in postnatal day 7 rodents, an effect that was attenuated by intraperitoneal administration of the iron chelator deferoxamine (294). Irp1 levels did not change in response to GMH-IVH (294).

#### Amyloid precursor protein

APP is an integral membrane protein found in various tissues and organs including the brain and spinal cord (295). While it does not directly handle iron, APP helps regulate iron homeostasis in neurons, BCECs, and other brain cells by stabilizing cell surface presentation of the iron exporter FPN1 (296–298). APP and Amyloid-Precursor Like Proteins have additional physiological functions in neurodevelopment, where they are thought to contribute to neurogenesis (299–304), neurite growth (305–314), and synaptogenesis (315–318).

Downstream proteolytic processing of APP where APP is cleaved by α-secretase can produce soluble amyloid precursor protein α (sAPPα) (319, 320). APP can also be cleaved by β-secretase and γ-secretase to produce the Aβ1-42 peptide and sAPPβ (321–325), which is a precursor to soluble amyloid-beta. In humans, CSF sAPP is a potential diagnostic biomarker for diseases like Alzheimer’s Disease and Multiple Sclerosis (316, 320, 326), and as a potential predictor of neurodevelopmental outcomes after neonatal GMH-IVH (327, 328).

##### Developmental time course of amyloid precursor protein in the brain

APP is expressed in the fetal and perinatal brain at various stages of neurodevelopment. In a study investigating App expression in embryonic day 8.5–13.5 mice, APP was identified in hindbrain and spinal cord motor neurons and cranial ganglia neurons as early as embryonic day 9.5 (299). APP expression increased to embryonic day 10.5, with continued increases in intensity and spread to the last time point studied (299). *App* mRNA was also identified in these brain regions at embryonic day 13.5, suggesting CNS-endogenous App production. Other studies have shown that *App* mRNA is expressed in the mouse brain at embryonic day 12, with subsequent increases up to 15-fold before it plateaus at postnatal day 10 (299). App has also been identified in radial glial cells in the fetal and neonatal mouse brain (300).

##### Amyloid precursor protein after neonatal GMH-IVH

Specific to neonatal IVH, CSF levels of sAPPα are significantly elevated after IVH-PHH in humans compared to control infants (328, 329), and furthermore CSF APP levels are associated with ventricular size after neonatal IVH (327). As APP is released by axonal injury (330–332), this finding may be related to axonal stretch in periventricular regions due to ventricular distention (327). Alternatively, as it is known that APP is upregulated in the presence of free iron (333), and that iron plays a key role in the pathogenesis of PHH after IVH (101), it is possible that APP reflects high brain iron levels after IVH in neonates that develop the most severe ventriculomegaly.

## Iron pathway and related proteins that have not been functionally characterized in the neonatal brain

**Figure 2:**
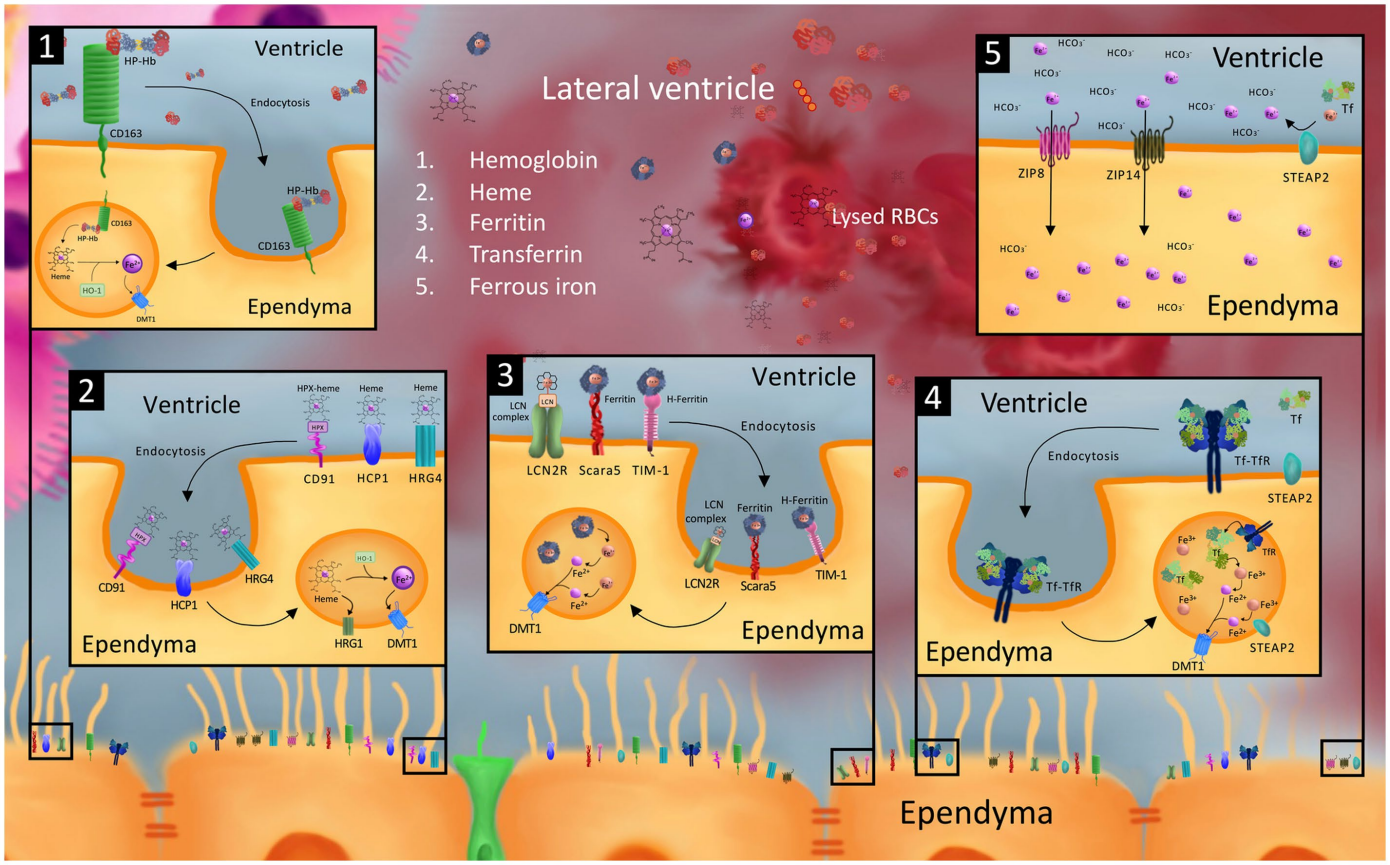
Model of possible cellular iron transporters that may be involved in iron uptake into the ependyma and choroid plexus (ChP) from the CSF after germinal matrix hemorrhage-intraventricular hemorrhage (GMH-IVH). While the exact mechanism of ependymal and choroid plexus uptake of CSF blood and blood breakdown products after GMH-IVH is unknown, it is possible that cellular iron transporters involved in blood breakdown product and iron uptake in other cells of the neonatal and adult brain, neonatal peripheral tissue, and other organs may be involved. Specifically, we model ependymal uptake of hemoglobin-haptoglobin (Hb-Hp) from the CSF via the CD163 receptor (1); heme-hemopexin (hpx) and heme via CD91, heme carrier protein 1 (HCP1), and heme responsive genes 4 and 1 (2); ferritin and Fe3 + −containing lipocalin 2 (LCN) complex via scavenger receptor class A member 5 (Scara5), T cell immunoglobulin and mucin domain containing protein 1 (TIM-1), and LCN2 receptor (LCN2R) (3); transferrin (Tf) via transferrin receptor (TfR) (4); and ferrous iron ions (Fe2+) via the zinc regulated transporter and iron regulated transporter like proteins 8 and 14 (ZIP8 and ZIP14) (5). Intracellular processing in the endosomal compartment to convert receptor-mediated endocytosed blood breakdown product compounds into Fe2+ ions for export into the cytosol via divalent metal transporter 1 (DMT1) and six-transmembrane epithelial antigen of the prostate protein 2 (STEAP2) converting Fe3+ ions into Fe2+ ions are also shown. To our knowledge, the majority of these transporters (including but not limited to ZIP8, ZIP14, Scara5, TIM-1, LCN2R, CD163, CD91, HCP1, HRG4) have not been identified on the ependyma, and are only modeled in this figure as playing a hypothetical role in transporting their corresponding blood breakdown/iron product to guide future investigations.

Beyond DMT1, TFR, and FPN1, the localization and developmental expression of few if any *cellular* iron handling proteins known to be important in iron handling in the adult brain and other tissues outside of the CNS have been reported in the neonatal brain. This section discusses the role of several additional iron handling proteins in peripheral tissues in the neonate, reviews their function in the adult brain, and proposes potential roles they may play in the neonatal brain. It is important to consider additional iron transporters in the neonatal brain as they may play key, therapeutically targetable roles in iron handling after GMH-IVH (Figure 2).

### Heme responsive gene 1

Heme Responsive Gene 1 (HRG1), also known as solute carrier family 48 member 1 (SLC48A1), is a transmembrane receptor involved in mediating cellular heme homeostasis. Initially identified as an Hrg4 paralog in the *C. elegans* genome (334), subsequent experiments in zebrafish demonstrated *Hrg1* mRNA expression throughout the embryonic CNS (334). Functional experiments revealed that Hrg1 has a role in neurodevelopment and erythropoiesis in the zebrafish embryo (334). Notably, Hrg1 knockdown in zebrafish embryos using antisense morpholinos induced hydrocephalus and yolk tube malformations (334), however the mechanism by which this happens is not clear. Unlike Hrg4 which transports heme from the extracellular environment into the cell, Hrg1 specifically functions to transport endocytosed heme from endosomal compartments into the cytosol (334, 335). *In vitro* experiments in mouse macrophages undergoing erythrophagocytosis show that Hrg1 transports heme from the phagolysosome out to the cytosol (336, 337).

In adult mammals, HRG1 expression has been identified in brain, kidney, heart, skeletal muscle tissue, liver, lung, placenta, and small intestine in lower quantities (334). Hrg1 protein expression has also been reported on the apical surface of murine renal cortex epithelial cells following neonatal hemolysis (335), and the apical membrane of porcine duodenal enterocytes in piglets fed hemoglobin-enriched diets (338).

Neither the role of HRG1 in the adult brain nor the specific patterns of HRG1 expression in the neonatal brain are well understood. The potential roles of HRG1 in heme transport in the neonatal brain, particularly in connection with cellular heme and iron overload after IVH, merits further investigation alongside other proteins in the HRG family including HRG-3 (339).

### Heme carrier protein 1

Like HRG1, heme carrier protein 1 (HCP1) is a transmembrane protein that transports heme in addition to folate and heme-hemopexin complexes. Also known as solute carrier family 46 member 1 (SLC46A1) and proton-coupled folate transporter (PCFT), Hcp1 was initially characterized on the brush-border membrane of murine duodenal enterocytes where it mediates heme uptake and intestinal heme transport (340). Subsequent studies have reported HCP1 protein and/or mRNA expression on human macrophages and cultured astrocytes (341, 342), and within the human retina and retinal pigment epithelium, and mouse duodenum, liver, and kidney (340, 343).

Within the CNS, variable levels of Hcp1 expression have been identified in the cortex and hippocampus of adult rats (344), and *in vitro* experiments demonstrated neuronal heme uptake via Hcp1 (344). HCP1 is also expressed on the basolateral surface of the neonatal and adult ChP as a proton symporter that mediates folate transport into the CSF (345). The role of ChP HCP1 in heme transport is not as well-explored.

### Lipocalin 2

Lipocalin 2 (LCN2) is an inducible protein secreted by a variety of cell types in the brain, liver, and uterus that mediates intercellular communication, innate immune responses to bacterial infections, and iron homeostasis. Specific to iron, LCN2 mediates transferrin-independent iron delivery and removal from cells by way of sequestering sidephores (346, 347), iron chelators that scavenge ferrous iron with high affinity and specificity. While LCN2 is not well-characterized in the neonatal brain, LCN2 secretion from neurons and glia has previously been reported to be inducible *in vitro* by exposure to amyloid-beta (348), and *in vivo* by hemoglobin (349), kainic acid (350), and other compounds. LCN2 is also known as neutrophil gelatinase-associated lipocalin (NGAL), 24p3, and p25, and more archaically siderocalin and uterocalin.

Lcn2 signaling in the adult brain can be upregulated by CNS pathology including spinal cord injury, autoimmune encephalomyelitis, and ICH, and is generally considered to have a role in mediating downstream neurotoxic effects (351–357). Lcn2 is expressed in astrocytes and ECs in the adult mouse brain after middle cerebral artery occlusion in a murine stroke model (358), while the Lcn2 receptor is expressed in neurons, astrocytes, and endothelial cells (358). Similarly, Lcn2 is involved in ischemic stroke reperfusion injury (358, 359) and increases in brain Lcn2 levels after stroke play a role in mediating subsequent brain injury by activating astrocytes (360). LCN2 is also upregulated after traumatic brain injury and has been shown to be a chemokine inducer in the murine adult CNS both *in vitro* and *in vivo* (361, 362).

In a mouse model of adult IVH, intraventricular hemoglobin induced Lcn2 upregulation and ventriculomegaly (349) where Lcn2-deficient mice were protected against hemoglobin-induced ventricular dilation, glial activation, and mortality (349). This finding mirrors a report of reduced white matter damage and decreased BBB disruption after SAH in Lcn2-deficient mice compared to wild-type controls (363). Lcn2 causes morphological changes in neuronal dendritic spines to decrease spine density and promote spine elimination in stress conditions (364), supporting its role in responding to adverse and/or noxious stimuli to result in inflammatory or neurologic injury.

LCN2 has been studied in peripheral tissues in developing and neonatal organisms. *In vitro* Lcn2 expression in embryonic day 13 ureteric bud cells derived from the developing rat kidney was reported to induce mesenchymal cell differentiation into epithelial cells (365). Lcn2 also functions as an iron delivery protein to transport and deliver iron to kidney epithelial progenitors and stroma cells during embryonic organogenesis *in vitro* (365). In humans, serum, urine, stool, and umbilical cord levels of LRP2 have been used as diagnostic and predictive markers for renal impairment/injury (366–372), necrotizing enterocolitis (373), sepsis (374), and other conditions in preterm neonates with acute and chronic pathologies. While LCN2 has not been reported in the neonatal brain, the Lcn2 receptor megalin is expressed in the neural tube and developing rat and mouse brain where it plays a role in neurodevelopment (375, 376).

### Scavenger receptor class A member 5

Scavenger receptor class A member 5 (SCARA5) belongs to a class of membrane receptors and can recognize and bind a variety of substrates including serum ferritin to mediate ferritin-bound non-transferrin iron uptake and delivery (377, 378). SCARA5 recognizes both H- and L-ferritin in a Ca^2+^-dependent manner and is expressed in the adult human and mouse retina (379, 380), the developing mouse kidney (377), and epithelial cells in the murine testis, bladder, trachea, adrenal glands, skin, lung, brain, and ovary (378). *In vitro* experiments in cells derived from human spleen report SCARA5 directly binds to and mediates the intracellular internalization of Von Willebrand Factor, a large multimer that plays an essential role in clotting and hemostasis (381).

While there have been few reports investigating cellular SCARA5 localization in the central nervous system, experiments from an *in vitro* model of the BBB show SCARA5 in brain endothelial cells mediates substrate uptake into brain endothelial cells to cross the BBB (382). Similar results have been reported *in vivo* in mice with Scara5-mediated ferritin uptake across the blood-retina barrier (379). Low-level Scara5 expression has been reported on cultured astrocytes derived from neonatal mouse brains (383). In adult humans, SCARA5 is highly enriched in the ChP, with additional expression in the cerebral cortex, basal ganglia, cerebellum, and spinal cord (384).

In a study using Mendelian randomization to identify biomarkers for stroke, SCARA5 levels were associated with a decreased risk of SAH, potentially implicating SCARA5 in baseline risk for hemorrhage (385). SCARA5 also had consistent but non-significant effects on ICH risk (385). It is not clear whether this effect is mediated by SCARA5’s interactions with ferritin, or alternatively its role in binding VWF. SCARA5 expression in the brain has not been investigated after adult or neonatal IVH.

### Hypoxia-inducible factor

Hypoxia-inducible factor (HIF) is a heterodimeric transcription factor which mediates the adaptive homeostatic response to hypoxia (386, 387). HIF has been studied as a link between iron homeostasis and erythropoiesis (388, 389). HIF binds to hypoxia-responsive elements (HREs) in the regulatory regions of target genes including genes crucial to iron homeostasis like *Tfr* (390), transferrin (391), ceruloplasmin (392), and *Hmox-1* (393).

While the role of HIF in pathologic conditions in the neonatal brain like hypoxic–ischemic injury are well-studied, its role in neonatal brain iron homeostasis in physiologic conditions is not well-explored. HIF subunits and their transcriptional pathways are essential regulators of iron homeostasis in the intestine (389, 394–396). *In vivo* studies in mice have reported intestinal *Dmt1* and *Fpn1* are direct transcriptional targets of HIF-2α (394–397), and disrupting Hif-2α signaling in the intestine leads to impaired iron absorption (394). As *Hif-1α*, *Hif-2α*, and *Hif-1β* are expressed in the developing brain and are crucial for healthy brain development (398, 399), HIF may be a targetable aspect of brain iron homeostasis to prevent iron overload after neonatal IVH.

### Zinc regulated transporter and Iron regulated transporter-like proteins

Zinc regulated and Iron regulated transporter-like proteins (ZIP) are a family of metal ion transporters encoded by the SLC39 gene that imports divalent metal ions including Zn^2+^, Fe^2+^, and Mn^2+^ into the cytoplasmic compartment of cells. ZIP8 and ZIP14 are closely related and are the most-studied ZIP proteins in the context of iron trafficking. Both proteins have been identified *in vitro* on human-derived BCECs that constitute the BBB and hippocampal neurons (57, 400), and Zip8 and Zip14 have separately been shown to mediate cellular NTBI uptake (57, 401, 402). Zip14 may also have additional roles in iron acquisition from endocytosed transferrin *in vitro* (403). Additional *in vitro* studies report Zip8 is localized at the cell surface of the neuronal soma and dendrites, where it co-localizes with Steap2 and Tfr, while Zip14 concentrates within the cytosol and nucleus (57). Of note, Zip8 expression has been identified in mouse neural progenitor cells both *in vitro* and *in vivo* at E14, with expression significantly decreasing after differentiation (404).

While ZIP-mediated transport of zinc and several other divalent metal ions is known to play a crucial role in healthy development and growth (405, 406), ZIP8 and ZIP14’s roles in iron transport in the neonatal brain are relatively underexplored. Likewise, while a ZIP8 missense variant in humans has been associated with cerebrovascular disease and ICH (407), ZIP8 and ZIP14 expression and localization in the CNS after ICH, SAH, and/or IVH is not well-understood. As Zip8 expression has been shown to be upregulated in cell iron overload in the retina, and knock down of Zip14 is able to prevent iron overload in hepatocytes in a mouse model of hereditary hemochromatosis (408), elucidating ZIP8 and ZIP14 expression patterns (1) in the developing brain, and (2) after hemorrhage represent potential next steps toward understanding ZIP8 and ZIP14 as potential therapeutic targets to prevent brain iron overload after neonatal IVH.

## Downstream pathways of blood breakdown product and iron overload after IVH

When physiologic mechanisms of iron transport and regulation fail to keep up with accumulating iron levels in the brain after IVH, ferrous iron may react with hydrogen peroxide to generate free radical oxygen species via the Fenton reaction. Hydrogen peroxide is widely available as a small-molecule messenger in the brain, with the mitochondria serving as the major intracellular site of hydrogen peroxide production (409, 410). An imbalance in ferrous iron levels can thus result in rapid and fulminant production of reactive oxygen species that overwhelm the brain’s antioxidant capabilities, leading to oxidative stress.

There are several mechanisms by which oxidative stress is hypothesized to lead to cellular toxicity. Reactive oxygen species can react with a variety of biological macromolecules including lipids, proteins, nucleic acids, and carbohydrates (411). The polyunsaturated fatty acyl side chains in polyunsaturated fatty acids which make up cellular membranes are particularly susceptible to damage via lipid peroxidation (412, 413). This process can disrupt cell and organelle integrity directly via peroxidation of the inner mitochondrial membrane phospholipid cardiolipin (412, 414–416), and indirectly by producing signaling molecules (i.e. those in the NF- *κ*B, mitogen-activated protein kinase, and protein kinase C signaling pathways) capable of inducing both intrinsic and extrinsic apoptotic pathways (415, 417, 418). Lipid peroxidation can also drive ferroptosis, a recently identified mechanism of iron-dependent oxidative stress which leads to non-apoptotic programmed cell death (419), however the precise mechanism by which lipid peroxidation and ferroptosis are connected is not well understood (420). Ferroptosis induction in the ChP has recently been identified as a potential mechanism of cell death after PHH (421, 422), and iron chelators like deferoxamine (DFX) are thought to inhibit lipid peroxidation (415, 423).

In addition to oxidative stress, IVH also induces neuroinflammation and innate neuroimmune activation (424, 425). A recent study using a rat model of IVH induced via intraventricular hemoglobin injection demonstrated acute increases in brain-wide cytokine production and microglia reactivity followed by localized oxidative stress in the white matter (424). Acute ChP and lateral ventricle ependyma inflammation via NF- *κ*B activation has also been reported in rats in response to intraventricular autologous blood (426), as well as inflammation mediated by the TLR4-dependent cytokine TNF-α (147, 427–429). Autologous blood injected into the ventricles of adult mice has also been shown to increase cytokine secretion at the ChP-CSF interface mediated by activation of ChP-associated macrophages (81). Unlike reactive oxygen species generation via the Fenton reaction and oxidative stress, the mechanisms of neuroinflammation after IVH have not specifically been linked to iron overload. While hemoglobin and iron released into the CSF after IVH can increase macrophage and resident microglia activation to facilitate HMOX-1-mediated heme degradation, thrombin, periredoxin 2, methemoglobin, and other blood and blood breakdown product components represent additional candidates (427, 428, 430–433).

## The role of iron in the pathogenesis of post-hemorrhagic hydrocephalus and brain injury following intraventricular hemorrhage

Approximately 30% of infants with high grade (grade III or IV) IVH develop PHH (14), an imbalance in the production and efflux of CSF resulting in symptomatic ventriculomegaly. While it is known that blood within the ventricles and brain is the primary risk factor for PHH after IVH, the specific etiology of PHH after IVH is unclear with various potential mechanisms (iron-mediated toxicity, impaired CSF dynamics, inflammation, CSF hypersecretion, ependymal denudation etc.) (12, 81, 424, 434–436).

Strahle et al. previously reported that intraventricular injection of hemoglobin and iron, but not the iron-deficient heme precursor Protoporphyrin IX, results in ventriculomegaly 24 h post-injection in rats (101). Clinical studies have reported elevated CSF non-protein-bound iron in preterm infants with posthemorrhagic ventricular dilation compared to control infants (16). Iron chelation with both peripheral and intraventricular deferoxamine has been shown to reduce ventriculomegaly after IVH in rats (101, 437–440). Together, these results suggest iron is linked to PHH pathogenesis, however the mechanism by which the two are connected is not clear.

One mechanism by which free iron in the CSF and brain parenchyma may lead to PHH is via its cytotoxic effects on specific structures that play key roles in maintaining homeostasis between CSF production and drainage. In one theory of PHH pathogenesis, iron-mediated ependymal and cilia dysfunction is hypothesized to hinder normal CSF circulation and lead to CSF accumulation. E1 ependymal cells, which are one of three subtypes of ependymal cells (E1–E3), line the ventricles and have motile cilia on their apical surface that are damaged and sloughed off by blood breakdown product release into the CSF (441–444). Primary cilium on E2 and E3 ependymal cells, which play a sensory role in detecting chemical and mechanical changes in the CSF (441, 445–447), may also be damaged by iron. The resultant cilia loss and ependymal denudation may (1) expose underlying cells to blood and CSF leading to edema and gliosis of subependymal tissue, (2) impair ventricle wall ependyma-ChP communication and feedback, and (3) alter the velocity, turbulence, and/or vorticity of CSF flow patterns (448). In combination, these effects may contribute to the onset of hydrocephalus by disrupting CSF homeostasis by way of altering CSF flow, however this hypothesis has recently been questioned in light of data suggesting cilia are not the primary drivers of CSF movement in the ventricular system in humans (434, 449–452).

Aberrant CSF production in response to iron-mediated ChP damage and ChP inflammation has been posited as an alternative mechanism of PHH. In a rodent model of neonatal IVH, intraventricular blood was reported to induce early ChP epithelial cell activation and transient increases in ChP Na^+^/K^+^/2Cl^−^ co transporter (NKCC1) expression and phosphorylation (436), both which have been linked to subsequent hydrocephalus development and/or increases in PHH severity (436, 453). A separate study also reported ChP transporter activation after IVH and the resultant CSF hypersecretion contribute to PHH onset (454), however it is not clear if this is a direct effect of iron cytotoxicity or if these effects are secondary to inflammation (454–456). Recently, a mechanism by which autologous blood may induce a TLR4-mediated inflammatory response at the ChP has been proposed (81), serving as a potential link between blood breakdown product release into the CSF in IVH, ChP inflammation, CSF hypersecretion, and the onset of PHH.

Other proposed mechanisms of PHH development implicate the complement cascade (457, 458); aquaporins 1, 4, and 5 upregulation (437, 459, 460); CSF pathway obstruction by blood breakdown products, blood clots, debris, or fibrosis secondary to inflammation (12, 461–463); and dysfunction of the blood–brain and blood-CSF barriers (464). Altogether, these proposed mechanisms fall under two major categories – CSF underdrainage and CSF overproduction (465). While it is not entirely clear how iron may be directly linked to either under- or overdrainage, it is clear that intraventricular iron is necessary, but not sufficient, to induce hydrocephalus after IVH. Other genetic factors may underlie development of hydrocephalus in response to IVH (466). Therefore, it will be important to consider how iron may contribute to both CSF under resorption and hypersecretion in tandem to best uncover the mechanism(s) by which iron may be linked to PHH onset.

Iron and blood-breakdown products have been implicated in the pathogenesis of not only PHH, but also direct injury to the brain. Intraventricular injection of hemoglobin in neonatal rats has previously been linked to neuronal degeneration in the hippocampus via the c-Jun N-terminal kinase (JNK) pathway (102). Exposure to intraventricular hemoglobin resulted in significant decreases in hippocampal volume, a finding which was reversable with iron chelation with deferoxamine (102). A separate study conducted in human neonates reported that very preterm infants with high-grade IVH and PHH had the smallest hippocampal volumes and the worst neurodevelopmental outcomes at 2 years of age compared to full-term infants and very preterm infants without brain injury (103). In conjunction with previous studies which demonstrated associations between smaller hippocampal size and worse cognitive and motor outcomes in humans (467, 468), these results together suggest that iron overload and toxicity after IVH may lead to hippocampal neuronal degeneration, which in turn leads to impaired neuromotor developmental outcomes. Furthermore, to directly relate intraventricular blood breakdown product levels with behavioral outcomes, intraventricular hemoglobin injection in rodents has been directly associated with an impaired acoustic startle response (437), a behavioral impairment which was prevented when rodents were treated with intraventricular deferoxamine at the time of IVH (437). Future studies should investigate the precise mechanisms by which iron and blood breakdown product exposure may lead to hippocampal neuron toxicity (ie. oxidative stress, ferroptosis) to identify targetable mechanisms for prophylactic treatment and prevention of IVH-induced brain injury and resultant neurodevelopmental effects.

## Therapeutic directions for neurological sequelae after GMH-IVH that target iron homeostasis in the neonatal brain

Despite the devastating long-term neurodevelopmental consequences and mortality risk of PHH, there are currently no curative or preventative solutions available. Up to 34% of very low birth weight infants with persistent ventricular dilation after IVH require surgical intervention for CSF diversion with a shunt (469). While CSF diversion remains the gold standard treatment for hydrocephalus, it is associated with short and long-term complications including shunt failure rates of 33% with a mean time to failure of 344 days (470). Endoscopic third ventriculostomy (ETV) with or withhout ChP cauterization (CPC) is an alternative treatment option however it does not have high success rates for the treatment of PHH (471–474).

In line with the hypothesis that iron plays a role in PHH pathogenesis, recent pre-clinical studies have investigated iron chelators as a possible treatment to prevent PHH after adult and neonatal IVH (101, 104, 437–440, 475). DFX is an iron chelator that sequesters free iron and has been used in the clinical setting to treat hemochromatosis (476, 477), thalassemia (478–480), and other iron-overload syndromes. Previous research in an adult rat model of IVH-PHH reported that consecutive intraperitoneal deferoxamine administered in 12-h intervals over 7 days starting 3 h post-IVH decreased CSF iron and brain ferritin levels and reduced the incidence of ventriculomegaly 7 and 28 days after the start of DFX treatment (438). Gao et al. also demonstrated intraventricular co-injection of deferoxamine with lysed RBCs markedly reduced ventricular enlargement compared to rats injected with lysed blood cells in a rat model of adult IVH (475). In neonatal rodents, intraventricular administration of deferoxamine at the time of hemorrhage in a neonatal rat model of hemoglobin-induced IVH delays the onset of PHH for 11 days and prevents long-term behavioral deficits (437). In addition, intraperitoneal administration of deferoxamine 2 h after IVH followed by *bis in die* intraperitoneal administration over 24 h reduced hemoglobin-induced ventricular enlargement (101). These findings have been replicated where deferoxamine reduced ventriculomegaly, improved motor and cognitive function, and prevented other neurological sequelae in a rodent model of neonatal GMH-IVH (439).

In addition to deferoxamine, the tetracycline antibiotic minocycline has also been shown to inhibit ferritin upregulation, reduce edema, and prevent hydrocephalus after hemorrhage induction in a rodent collagenase model of GMH by way of chelating iron to reduce brain iron overload (104). Hemoglobin sequestration compounds including haptoglobin and α_1_-microglobulin have also been explored as neuroprotective treatment options to attenuate the neurotoxic and inflammatory consequences of excess hemoglobin released in IVH (481, 482). Intraventricular administration of erythropoietin and melatonin, hormones with roles in the survival and maturation of developing neural cells and in reducing neuroinflammation and oxidative stress, has also been shown to preserve neonatal neurodevelopment and prevent ventriculomegaly in a rat model of neonatal chorioamnionitis-IVH (442). The efficacy of melatonin and erythropoietin in combination to prevent PHH after IVH is also currently being evaluated in a clinical trial.

### Potential therapeutic avenues that have not yet been explored

Beyond ongoing research into the efficacy of these and other therapeutic strategies for preventing PHH after IVH, other potential treatment options that address additional aspects of IVH-PHH pathophysiology and warrant further investigation include experimentally up/down regulating iron transporters on ependyma and other brain cells, adapting drugs used clinically for other disorders causing iron overload, and combining these strategies with techniques that improve the delivery of drugs to the brain.

Specifically, one of the cellular iron transporters we discussed in this manuscript, FPN1, may represent a particularly intriguing candidate for experimental up- or downregulation after IVH as the only known non-heme iron exporter that has been identified in mammals. In adult Lewis and LEWzizi rats, Fpn1 is localized to the apical surface of ChP epithelial cells and the basolateral surface of ependymal cells (483). FPN1 expression patterns along the ventricular system of neonates are not as clear and should be characterized for use as a potential target against iron overload in the ependyma, ChP, and/or brain parenchyma.

In addition to targeting the expression of iron transporters to prevent CSF iron from accumulating in cells and resulting in cellular iron overload, facilitating iron removal from the CSF with drugs used clinically for systemic iron overload (ie. previously studied examples of DFX and minocycline) represents an alternative therapeutic avenue. Beyond DFX and minocycline, adapting drugs which have been shown to reverse hemochromatosis and brain siderosis may present alternative options for iron chelation after IVH. For example, Hudson *et al* and Hale *et al* previously showed in mice that whole animal and intestine-specific genetic knockout of the lithium-sensitive enzyme bisphosphate 3′-nucleotidase (Bpnt1) enzyme leads to iron deficiency anemia and can rescue hemochromatosis (484, 485). Lithium may thus have potential to reduce iron levels after IVH and can be further investigated as a therapeutic agent; however, lithium may have off target effects.

Finally, the BBB presents a major challenge to brain iron chelation that is not present in treatment of systemic disorders of iron overload. However the BBB, in conjunction with the blood-CSF barrier, can be targeted with techniques that improve the delivery of drugs to the brain. Combining drug candidates with nanoparticles to circumvent the BBB (486–488), using focused ultrasound to open localized regions of the BBB (489–495), and other evolving drug-delivery strategies all represent solutions that may improve the efficacy, efficiency, and breadth of future investigations into the potential therapeutic avenues we describe in this manuscript and should be taken advantage of going forward.

## Conclusion

Maintaining the delicate balance in brain iron levels is vital for healthy brain development and is facilitated by a variety of iron handling proteins with specialized roles in brain and cellular iron import and export. GMH-IVH results in major disruptions to brain iron homeostasis during the neonatal time period through release of blood and blood breakdown products into the ventricular system. IVH is thought to be linked to PHH via iron overload-mediated pathogenesis, however the specific mechanisms by which this occurs are not clear. Determining the iron-mediated mechanisms by which IVH results in PHH is complicated due to a relative paucity of understanding for the function of baseline iron transport proteins in the developing brain. Identifying additional iron transport proteins in the neonatal brain and investigating their role in iron handling after neonatal IVH may lead to additional treatment strategies to address and prevent iron-mediated neurological sequelae of GMH-IVH including PHH.

## Author contributions

SP: Writing – original draft, Writing – review & editing. AH: Writing – review & editing. ML: Writing – original draft. DR: Writing – original draft. TG: Writing – original draft. BS: Writing – original draft. KM: Writing – original draft. JS: Conceptualization, Writing – original draft, Writing – review & editing.
