# Supplementary material for: Iron homeostasis and post-hemorrhagic hydrocephalus: a review
Source: Front Neurol. 2024 Jan 12;14:1287559. doi: 10.3389/fneur.2023.1287559 (PMC10811254; doi:10.3389/fneur.2023.1287559)
Supplement: Supplementary file 1 [file Table_1.DOCX]

|  |  | **Developmental time course (prenatal)** | **Developmental time course (postnatal)** | **Roles after IVH/PHH** | **References** |
| --- | --- | --- | --- | --- | --- |
| Extracellular iron transporters and scavengers identified in the neonatal brain |  |  |  |  |  |
|  | Transferrin | CSF: peaks at 18 weeks’ gestation.*** | Peaks at birth and declines over postnatal weeks 2-3. Stabilizes at postnatal day 24 to stay constant through life.**, ***  BCECs: Expressed throughout development.  CSF: Decreases from birth to postnatal day 10***  Oligodendrocytes: Onset and increase of transferrin synthesis during postnatal days 10-25.*** | Longitudinal increases in CSF transferrin after human neonatal IVH-PHH are associated with improved cognitive outcomes at 2 years of age.  No significant differences in CSF transferrin after low- and high-grade IVH compared to human neonates without IVH. | [1–11] |
|  | Haptoglobin | Neurons: Expressed at 6-8 weeks’ gestation. Decreases to variable levels at 9-22 weeks. Rises at 25-36 weeks.*  BCECs: variable expression at 6-10 weeks’ gestation. No expression after 14 weeks.* | Low compared to fetal development.* | Intraventricular haptoglobin after IVH in rabbits attenuates hemoglobin-induced inflammation, cytotoxicity, and structural damage.  No significant differences in CSF haptoglobin between human neonates with and without IVH or PHH. | [12–15] |
|  | Hemopexin | Neurons: Expressed from 3-36 weeks’ gestation.* |  | CSF hemopexin is not elevated after IVH-PHH in human neonates.  Only iron scavenger that is increased between temporary and permanent CSF diversion after IVH-PHH in human neonates.  Ventricle size after IVH in human neonates is inversely correlated with CSF hemopexin levels. | [1,14] |
|  | Ceruloplasmin | Neurons: Expressed from 14-18 weeks’ gestation, but not at 25 weeks.*  Glia: Expressed weakly from 14-22 weeks’ gestation.*  Anchored form is expressed at embryonic day 12.5, while diffusible form is not expressed until embryonic day 17.5.** | Increasing expression from postnatal day 1 to postnatal day 7, before subsequently decreasing.**  Astrocytes: expressed at postnatal day 3.*** | No significant differences in CSF ceruloplasmin after neonatal IVH-PHH in humans.  No significant differences in CSF ceruloplasmin between temporary and permanent CSF diversion. | [1,13,14,16] |
| Membrane iron transporters and scavengers identified in the neonatal brain |  |  |  |  |  |
|  | Transferrin  receptor (TfR) |  | BCECs: Expression peaks in postnatal week 2 between postnatal days 10 and 21.***  Neurons: Weak expression seen at postnatal day 5, but robust expression is not observed until postnatal day 15, with a peak at postnatal weeks 3-4.***  ChP ependymal cells: Little to no expression at postnatal days 5-10, but robust expression by postnatal day 15.*** | Not well understood. | [17,18] |
|  | Divalent metal transporter 1 (DMT1) |  | mRNA expression at postnatal day 3, with additional increasing expression in the cortex, hippocampus, striatum, and substantia nigra from postnatal weeks 1-3.***  ChP ependymal cells, glia, and neurons: Variable expression from postnatal days 5-10, which increases to robust expression by postnatal day 15.*** | Not well understood. | [18–21] |
|  | Ferroportin 1 (FPN1) | Expression across the central nervous system was previously reported*** | BCECs: expressed at postnatal day 0, with decreasing expression from postnatal days 1-8.***  Parenchyma: Increase with age, with the lowest expression seen at postnatal week 1. Increasing expression from postnatal weeks 1-9, with subsequent decrease from postnatal weeks 10-28. | Not well understood. | [22–24] |
|  | Low-density lipoprotein receptor-related protein 1 (LRP1) | Radial glia: Expressed at embryonic days 13.5-18.** | Brain expression peaks during postnatal development. Stable expression in glia, neuroblasts, and neurons through development and adulthood.**  Oligodendrocyte precursor cells: Percentage of oligodendrocyte precursor cells expressing Lrp1 increases across embryonic and postnatal development, with nearly ubiquitous expression in adulthood.** | Not well understood. | [25,26] |
| Intracellular iron transporters and scavengers identified in the neonatal brain |  |  |  |  |  |
|  | Ferritin | Glia: expressed from 6-36 weeks’ gestation, with increasing levels from 19-22 weeks’ gestation.* | Brain expression initially peaks at postnatal day 2 and decreases over postnatal weeks 1-2. Levels begin to rise again at postnatal day 17, and stabilizes at levels similar to those seen at postnatal day 2 by postnatal week 11.*** | Longitudinal decreases in CSF ferritin between temporary and permanent CSF diversion after PHH in human neonates are associated with improved scores on cognitive and motor aspects of the Bayley III examination at 2 years of age.  Larger ventricle size at the time of permanent CSF diversion is associated with higher levels of CSF ferritin in human neonates.  Elevated levels of CSF ferritin associated with early and severe ventriculomegaly after IVH in human neonates.  Increase in number of ferritin-positive cells in periventricular areas and hippocampus after IVH in mice. | [1,2,13,14,27–29] |
|  | Heme oxygenase 1 (HMOX-1) |  | mRNA and protein levels are high at postnatal days 1 and 3 and decline out to adulthood.*** | Increase in hippocampus, cortex, and periventricular expression after neonatal GMH-IVH in mice. | [30,31] |
| Related proteins important in neonatal brain iron homeostasis |  |  |  |  |  |
|  | Iron regulatory protein 1 (IRP1) | Low mRNA expression (relative to iron regulatory protein 2).* | Variable expression from postnatal days 5-10, before increasing at postnatal day 15.*** | Not well understood.  No change after GMH-IVH in rodents. | [18]  [18,32] |
|  | Iron regulatory protein 2 (IRP2) | High mRNA expression (relative to iron regulatory protein 1).* | Variable expression from postnatal days 5-10, before increasing at postnatal day 15.*** | Decrease in expression 1-5 days after neonatal GMH-IVH in postnatal day 7 rodents. | [18,32] |
|  | Amyloid precursor protein (APP) | Protein expression across embryonic days 8.5-13.5, with increasing expression from embryonic days 10.5-13.5** | mRNA expression that increases from embryonic day 12 to postnatal day 10.** | Significantly elevated in the CSF after neonatal IVH in humans.  CSF APP levels associated with ventricular size after neonatal IVH. | [33–36] |

Supplementary Table 1. Pre- and post-natal developmental expression of iron-handling and iron-related proteins in the brain. * indicates findings derived from experiments using human tissue, ** mouse tissue, and *** rat tissue. CD163, STEAP, TIM, and hepcidin were excluded from the table because their developmental expression profiles are not as well-understood. Abbreviation: CSF, cerebrospinal fluid; IVH, intraventricular hemorrhage; PHH, posthemorrhagic hydrocephalus.

**References**

1. Strahle JM, Mahaney KB, Morales DM, Buddhala C, Shannon CN, Wellons JC, et al. Longitudinal CSF Iron Pathway Proteins in Posthemorrhagic Hydrocephalus: Associations with Ventricle Size and Neurodevelopmental Outcomes. Ann Neurol. 2021;90.

2. Jane A, Roskams I, Connor JR. Iron, transferrin, and ferritin in the rat brain during development and aging. J Neurochem. 1994;63.

3. Connor JR, Fine RE. Development of transferrin‐positive oligodendrocytes in the rat central nervous system. J Neurosci Res. 1987;17.

4. Markelonis GJ, Oh TH, Dion TL, Bregman BS, Pugh MA, Royal GM, et al. Localization of transferrin within the developing vertebrate nervous system. Rev Neurol (Paris). 1988.

5. Oh TH, Markelonis GJ, Royal GM, Bregman BS. Immunocytochemical distribution of transferrin and its receptor in the developing chicken nervous system. Developmental Brain Research. 1986;30.

6. Reynolds ML, Møllgård K. The distribution of plasma proteins in the neocortex and early allocortex of the developing sheep brain. Anat Embryol (Berl). 1985;171.

7. Toran-allerand CD. Coexistence of α-fetoprotein, albumin and transferrin immunoreactivity in neurones of the developing mouse brain. Nature. 1980;286.

8. Møllgård K, Jacobsen M, Jacobsen GK, Clausen PP, Saunders NR. Immunohistochemical evidence for an intracellular localization of plasma proteins in human foetal choroid plexus and brain. Neurosci Lett. 1979;14.

9. Dion TL, Markelonis GJ, Oh TH, Bregman BS, Pugh MA, Hobbs SL, et al. Immunocytochemical localization of transferrin and mitochondrial malate dehydrogenase in the developing nervous system of the rat. Dev Neurosci. 1988;10.

10. Møllgård K, Reynolds ML, Jacobsen M, Dziegielewska KM, Saunders NR. Differential immunocytochemical staining for fetuin and transferrin in the developing cortical plate. J Neurocytol. 1984;13.

11. Mllgård K, Stagaard M, Saunders NR. Cellular distribution of transferrin immunoreactivity in the developing rat brain. Neurosci Lett. 1987;78.

12. Gram M, Sveinsdottir S, Cinthio M, Sveinsdottir K, Åkerström B, Hansson S, et al. Extracellular Haemoglobin - Mediator of Structural Damage, Cell Death, Oxidative Stress and Inflammation in the Choroid Plexus Following Preterm Intraventricular Haemorrhage. Free Radic Biol Med. 2014;76.

13. Mahaney KB, Buddhala C, Paturu M, Morales D, Limbrick DD, Strahle JM. Intraventricular Hemorrhage Clearance in Human Neonatal Cerebrospinal Fluid: Associations with Hydrocephalus. Stroke. 2020;

14. Møllgård K, Dziegielewska KM, Saunders NR, Zakut H, Soreq H. Synthesis and localization of plasma proteins in the developing human brain. Integrity of the fetal blood-brain barrier to endogenous proteins of hepatic origin. Dev Biol. 1988;128.

15. Dziegielewska KM, Saunders NR, Schejter EJ, Zakut H, Zevin-Sonkin D, Zisling R, et al. Synthesis of plasma proteins in fetal, adult, and neoplastic human brain tissue. Dev Biol. 1986;115.

16. Ducharme P, Zarruk JG, David S, Paquin J. The ferroxidase ceruloplasmin influences Reelin processing, cofilin phosphorylation and neuronal organization in the developing brain. Molecular and Cellular Neuroscience. 2018;92.

17. Moos T, Oates PS, Morgan EH. Expression of the neuronal transferrin receptor is age dependent and susceptible to iron deficiency. Journal of Comparative Neurology. 1998;398.

18. Siddappa AJM, Rao RB, Wobken JD, Leibold EA, Connor JR, Georgieff MK. Developmental changes in the expression of iron regulatory proteins and iron transport proteins in the perinatal rat brain. J Neurosci Res. 2002;68.

19. Williams K, Wilson MA, Bressler J. Regulation and developmental expression of the divalent metal-ion transporter in the rat brain. Cell Mol Biol (Noisy-le-grand). 2000;46.

20. Knutson M, Menzies S, Connor J, Wessling-Resnick M. Developmental, regional, and cellular expression of SFT/UbcH5A and DMT1 mRNA in brain. J Neurosci Res. 2004;76.

21. Moos T, Skjoerringe T, Gosk S, Morgan EH. Brain capillary endothelial cells mediate iron transport into the brain by segregating iron from transferrin without the involvement of divalent metal transporter 1. J Neurochem. 2006;98.

22. Burdo JR, Menzies SL, Simpson IA, Garrick LM, Garrick MD, Dolan KG, et al. Distribution of Divalent Metal Transporter 1 and Metal Transport Protein 1 in the normal and Belgrade rat. J Neurosci Res. 2001;66.

23. Yang WM, Jung KJ, Lee MO, Lee YS, Lee YH, Nakagawa S, et al. Transient expression of iron transport proteins in the capillary of the developing rat brain. Cell Mol Neurobiol. 2011;31.

24. Jiang DH, Ke Y, Cheng YZ, Ho KP, Qian ZM. Distribution of ferroportin1 protein in different regions of developing rat brain. Dev Neurosci. 2002;24.

25. Hennen E, Safina D, Haussmann U, Wörsdörfer P, Edenhofer F, Poetsch A, et al. A LewisX glycoprotein screen identifies the low density lipoprotein receptor-related protein 1 (LRP1) as a modulator of oligodendrogenesis in mice. Journal of Biological Chemistry. 2013;288.

26. Auderset L, Cullen CL, Young KM. Low density lipoprotein-receptor related protein 1 is differentially expressed by neuronal and glial populations in the developing and mature mouse central nervous system. PLoS One. 2016;11.

27. Mahaney KB, Buddhala C, Paturu M, Morales DM, Smyser CD, Limbrick DD, et al. Elevated cerebrospinal fluid iron and ferritin associated with early severe ventriculomegaly in preterm posthemorrhagic hydrocephalus. J Neurosurg Pediatr. 2022;30.

28. Harrison PM, Arosio P. The ferritins: Molecular properties, iron storage function and cellular regulation. Biochim Biophys Acta Bioenerg. 1996.

29. Han J, Day JR, Connor JR, Beard JL. H and L ferritin subunit mRNA expression differs in brains of control and iron-deficient rats. Journal of Nutrition. 2002;132.

30. Strahle JM, Garton T, Bazzi AA, Kilaru H, Garton HJL, Maher CO, et al. Role of Hemoglobin and Iron in hydrocephalus after neonatal intraventricular hemorrhage. Neurosurgery. 2014;75.

31. Zhao H, Wong RJ, Nguyen X, Kalish F, Mizobuchi M, Vreman HJ, et al. Expression and regulation of heme oxygenase isozymes in the developing mouse cortex. Pediatr Res. 2006;60.

32. Samaniego F, Chin J, Iwai K, Rouault TA, Klausner RD. Molecular characterization of a second iron-responsive element binding protein, iron regulatory protein 2. Structure, function, and post- translational regulation. Journal of Biological Chemistry. 1994;269.

33. Morales DM, Silver SA, Morgan CD, Mercer D, Inder TE, Holtzman DM, et al. Lumbar cerebrospinal fluid biomarkers of posthemorrhagic hydrocephalus of prematurity: Amyloid precursor protein, soluble amyloid precursor protein α, and L1 cell adhesion molecule. Neurosurgery. 2017;80.

34. Morales DM, Townsend RR, Malone JP, Ewersmann CA, Macy EM, Inder TE, et al. Alterations in protein regulators of neurodevelopment in the cerebrospinal fluid of infants with posthemorrhagic hydrocephalus of prematurity. Molecular and Cellular Proteomics. 2012;11.

35. Morales DM, Holubkov R, Inder TE, Ahn HC, Mercer D, Rao R, et al. Cerebrospinal fluid levels of amyloid precursor protein are associated with ventricular size in post-hemorrhagic hydrocephalus of prematurity. PLoS One. 2015;10.

36. Salbaum JM, Ruddle FH. Embryonic expression pattern of amyloid protein precursor suggests a role in differentiation of specific subsets of neurons. Journal of Experimental Zoology [Internet]. 1994 [cited 2023 Jun 24];269:116–27. Available from: https://onlinelibrary.wiley.com/doi/full/10.1002/jez.1402690205
